# Together or Alone: Evaluating the Pathogen Inhibition Potential of Bacterial Cocktails against an Amphibian Pathogen

**DOI:** 10.1128/spectrum.01518-22

**Published:** 2023-01-31

**Authors:** Alexandra Alexiev, Melissa Y. Chen, Timothy Korpita, Andrew M. Weier, Valerie J. McKenzie

**Affiliations:** a Department of Ecology & Evolutionary Biology, University of Colorado, Boulder, Colorado, USA; Chinese Academy of Sciences

**Keywords:** *Anaxyrus boreas boreas*, *Janthinobacterium lividum*, *Batrachochytrium dendrobatidis*, boreal toad, multistrain, probiotics

## Abstract

The amphibian fungal skin disease *Batrachochytrium dendrobatidis* (*Bd*) has caused major biodiversity losses globally. Several experimental trials have tested the use of Janthinobacterium lividum to reduce mortality due to *Bd* infections, usually in single-strain amendments. It is well-characterized in terms of its anti-*Bd* activity mechanisms. However, there are many other microbes that inhibit *Bd in vitro*, and not all experiments have demonstrated consistent results with J. lividum. We used a series of *in vitro* assays involving bacterial coculture with *Bd* lawns, bacterial growth tests in liquid broth, and *Bd* grown in bacterial cell-free supernatant (CFS) to determine: (i) which skin bacteria isolated from a locally endangered amphibian, namely, the Colorado boreal toad (*Anaxyrus boreas boreas*), are able to inhibit *Bd* growth; (ii) whether multistrain combinations are more effective than single-strains; and (iii) the mechanism behind microbe-microbe interactions. Our results indicate that there are some single strain and multistrain probiotics (especially including strains from Pseudomonas*, Chryseobacterium,* and *Microbacterium*) that are potentially more *Bd*-inhibitive than is J. lividum alone and that some combinations may lead to a loss of inhibition, potentially through antagonistic metabolite effects. Additionally, if J. lividum continues being developed as a wild boreal toad probiotic, we should investigate it in combination with *Curvibacter CW54D,* as they inhibited *Bd* additively and grew at a higher rate when combined than did either alone. This highlights the fact that combinations of probiotics function in variable and unpredictable ways as well as the importance of considering the potential for interactions among naturally resident host microbiota and probiotic additions.

**IMPORTANCE** *Batrachochytrium dendrobatidis* (*Bd*) is a pathogen that infects amphibians globally and is causing a biodiversity crisis. Our research group studies one of the species affected by *Bd*, namely, the Colorado boreal toad (*Anaxyrus boreas boreas*). Many researchers focus their studies on one probiotic bacterial isolate called Janthinobacterium lividum, which slows *Bd* growth in lab cultures and is currently being field tested in Colorado boreal toads. Although promising, J. lividum is not consistently effective across all amphibian individuals or species. For Colorado boreal toads, we addressed whether there are other bacterial strains that also inhibit *Bd* (potentially better than does J. lividum) and whether we can create two-strain probiotics that function better than do single-strain probiotics. In addition, we evaluate which types of interactions occur between two-strain combinations and what these results mean in the context of adding a probiotic to an existing amphibian skin microbiome.

## INTRODUCTION

The rise of fungal emerging infectious diseases in recent decades is a threat to humans, agriculture, and wildlife, causing die-offs and extinctions globally (1). Fungal pathogens can be difficult to manage because few treatments are available and because they evolve resistance to antimicrobial drugs (1–4). One mitigation strategy is the use of probiotics, or potentially beneficial microbes associated with a host. Probiotics use a variety of mechanisms to keep pathogen loads low (e.g., preventing pathogen adhesion, supporting the host immune response, etc.) (5–8). These methods have been employed successfully in several industries, including human medicine, agriculture, and wildlife conservation (5, 8).

Probiotic strategies have previously been explored as a mitigation tool against amphibian chytridiomycosis, an infection caused by *Batrachochytrium dendrobatidis* (*Bd*) (5, 9–15). *Bd* has contributed to the decline of more than 500 amphibian species worldwide (16), and it is challenging to manage due to its rapid spread and evolution (17, 18). Researchers have considered various aspects of the biotic and abiotic factors that influence the success of probiotics (10, 19–25), and they have also cultured and tested *Bd*-inhibitive probiotic candidates *in vitro*, *in vivo*, and *in situ* (9–15).

There are many possible probiotic targets; however, the bacterium Janthinobacterium lividum is the most commonly studied (12, 13, 26–29). Janthinobacterium lividum was initially found to be ubiquitous on red-backed and four-toed salamanders, and it was able to inhibit *Bd* growth in culture (30, 31). It was subsequently isolated from the skin of different amphibian species, and its mechanism of *Bd* inhibition is well-characterized (12, 13, 27–29). Janthinobacterium lividum produces multiple *Bd* inhibitory metabolites, including violacein and indole-3-carboxaldehyde, which reduce *Bd* growth (27–29). The application of J. lividum to amphibians has seen some success (12–15); however, it has not always proven effective in some species, and many other *Bd*-inhibitive bacterial strains exist on amphibians (26, 32, 33). For example, past work in Panamanian amphibians found that the genera *Stenotrophomonas*, *Aeromonas*, and Pseudomonas had the highest proportions of anti-*Bd* isolates present, compared to those of other phylogenetic groups (32). It is also possible that other microbes might help J. lividum integrate into the community effectively and persist over time. Janthinobacterium lividum is part of the Burkholderiales group, which also contains other anti-*Bd* bacteria as well as taxa that are at high abundance on the skins of some amphibians (26, 34, 35). *In vitro* studies have found that combinations of anti-*Bd* bacterial isolates can be more successful than single-strains at inhibiting *Bd* (36–40). Thus, our work investigates how the J. lividum strain BTP, which is specifically found on this host, and other skin-associated bacteria interact with *Bd* on the Colorado boreal toad (*Anaxyrus boreas boreas*), which is a high-elevation wetland amphibian that is locally endangered in the Rocky Mountains due to *Bd* (41–44). Research on this species to date has prioritized the use of next-generation sequencing and *in vivo* treatments to develop an effective probiotic bioaugmentation strategy for boreal toads (12, 34, 35, 45–47). Adapting probiotic treatments to field conditions in wild populations remains as ongoing work.

Despite its increasingly widespread use, most probiotic research does not explore the mechanisms behind successful or unsuccessful trials alike (5, 8, 48). Our lack of mechanistic understanding could be a reason for the wide variation in how some host systems respond to probiotic treatments (49). The difference in treatment effectiveness could result from individual hosts harboring different, often already-established, communities, that then react in a variety of ways when a probiotic is introduced (e.g., via a priority effect) (50, 51). There are a number of additional possible mechanisms of pathogen inhibition that could be employed by single strain or multistrain bacterial mixtures. Such mechanisms include, but are not limited to, (i) a form of facilitation, wherein two microbes aid one another to affect *Bd* growth negatively and (ii) additive, synergistic, or antagonistic metabolite-induced inhibition (36, 37). Additive inhibition occurs when the inhibition caused by two metabolites equals their sum when they are combined. Synergistic inhibition occurs when the inhibition caused by two metabolites is more than their sum when combined. Antagonistic inhibition occurs when the inhibition of two metabolites is less than their sum when combined. A further benefit to identifying partners for a target probiotic strain is that it may allow strains to form the interactions necessary to integrate into the community effectively and persist over time.

Overall, we know little about the mechanisms by which wildlife probiotics act against pathogens in single strain or multistrain combinations. An exception is J. lividum. However, it is possible that other microbes or combinations of microbes could inhibit *Bd* more effectively. Using the Colorado boreal toad system, we aimed to answer the following questions:

Q1: Are there toad skin microbes that inhibit *Bd* other than J. lividum (*str. BTP*)?

Q2: Are two-strain combinations of microbes more inhibitive than single-strain treatments?

Q3: How do *Bd*-inhibiting microbes interact, especially given that we add probiotics to an existing microbiome? In particular, how do J. lividum (*str. BTP*) and other Burkholderiales interact?

**FIG 1:**
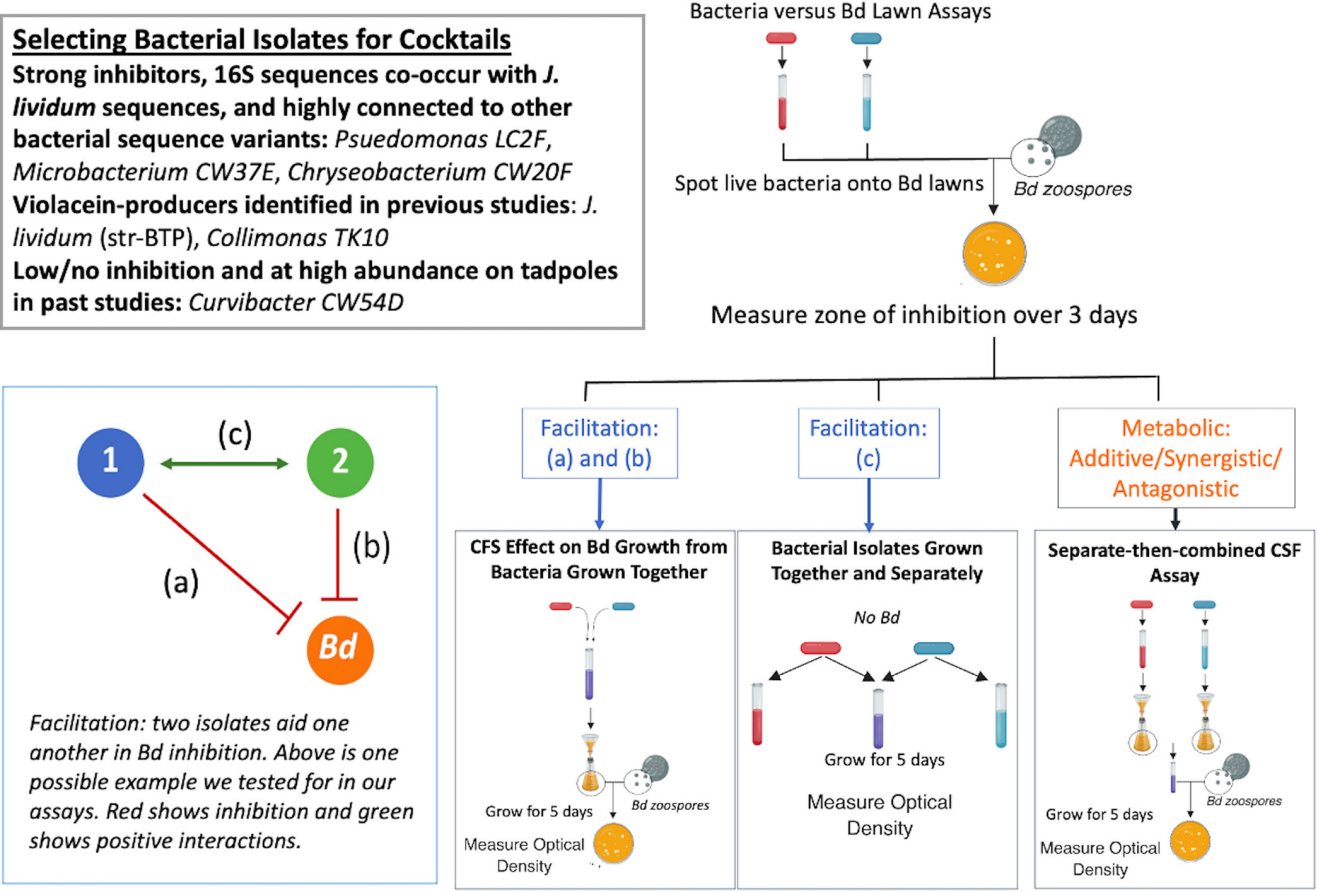
The overall workflow used to produce the data. Once bacteria were isolated and identified, we used a bacterial isolate versus *Bd* lawn coculture assay to determine which had *Bd*-inhibitive abilities. Of these, we chose a subset to further investigate the activity of cocktail mixtures involving two bacterial isolates. Using three different assays, we examined evidence for either antagonistic interactions between the isolates or facilitative interactions that could be additive or synergistic. Facilitation may involve three types of microbial interactions, and these are shown in the conceptual box at the bottom left of the figure, where isolates each inhibit *Bd* (a and b), and/or one isolate may confer benefits to the other isolate, thereby resulting results in the enhanced inhibition of *Bd* (c). Panels a, b, and c represent the facilitation interactions that were tested using the respectively labeled assays. Note that no *Bd* was used in the assay testing the facilitation interaction in panel c because this assay examines the effect of each isolate on the other’s growth to infer indirect facilitation.

To approach these questions, we conducted *in vitro* experiments with microbes. We isolated bacteria from wild boreal toads, identified them using sequencing, and challenged each isolate against *Bd* in a coculture assay. We then tested a selection of single strain and multistrain probiotic candidates using three different assays, wherein we examined evidence for either antagonistic interactions between the isolates or facilitative interactions, which could subsequently be additive or synergistic (Fig. 1). We define facilitation in the context of *Bd* inhibition as being when two isolates aid each other in the inhibition of *Bd*, and we tested whether the specific interactions shown in the model of facilitation in Fig. 1 were present. The first assay quantified whether the cell-free supernatant (CFS) from cocktails of bacterial isolates grown together effectively inhibited *Bd* growth, (see interactions [a] and [b] in Fig. 1). A second assay tested whether bacterial isolates had an effect on each other’s growth in the absence of *Bd* so as to test the potential of indirect facilitation of one bacterial isolate on another (see interaction [c] in Fig. 1). A third assay used an approach in which where bacterial isolates were grown separately and then had their CFS combined to examine the effect on *Bd* growth. Taken together, the results of these three assays allow us to disentangle the evidence for either antagonistic or facilitative interactions between different bacterial isolates and *Bd*.

## RESULTS

### Isolate selection.

From 125 candidate isolates, we selected 6 isolates to include in mechanistic assays in order to make further testing tractable in a controlled lab setting. We report genus-level, Sanger-sequence-based taxonomic identities throughout this paper when discussing the isolates used in the experiments described (Table S1). These include Pseudomonas *LC2F* sp., which have frequently been reported on other amphibians, often inhibit *Bd* (26, 32), and also co-occurred with most of the other sequence variants in previous 16S data (Table S2). *Chryseobacterium CW20D* have been reported from both captive and wild animals (12), and they have co-occurred with many other sequence variants (statistics are shown in Table S1). *Microbacterium* sp. and Pseudomonas sp. have been found in association with *Chryseobacterium* sp. in past studies of other systems (52–54). All three of these isolates were the most highly inhibitive of their genus of *Bd* lawns (Table 1). Three microbes from the order Burkholderiales (*Collimonas TK10*, *Curvibacter CW54D*, and J. lividum [*str. BTP*]), were selected for the mechanistic assays because they are commonly found on amphibians (34, 35) and because they frequently co-occurred with many other bacterial taxa in a co-occurrence analysis (Table S2). *Collimonas TK10* and J. lividum (*str. BTP*) were both chosen because they produce violacein and have been isolated previously from boreal toads as parts of separate, ongoing projects (27–29, 55). *Curvibacter CW54D* was chosen because its Sanger sequence is a strong match for the 16S sequence of an unknown Burkholderiales that is highly abundant in previously collected 16S data from boreal toad tadpole skin (34, 35, 56).

**TABLE 1:** Growth inhibition, measured as the diameter of the clearing zone in the *Bd* lawn, for each isolate tested on the *Bd* lawn assay (mean of duplicate measurements)^*a*^

Isolate name in manuscript	Mean growth inhibition (diam in mm)	SE (mm)
*Microbacterium CW37E*	5.5	0.5
Pseudomonas *LC2F*	10	0
*Chryseobacterium CW20D*	5	0
*Curvibacter CW54D*	0	0

a Trials involved growing each bacterial isolate either on or without a Bd lawn.

### CFS effect on *Bd* growth from bacteria grown together.

**FIG 2:**
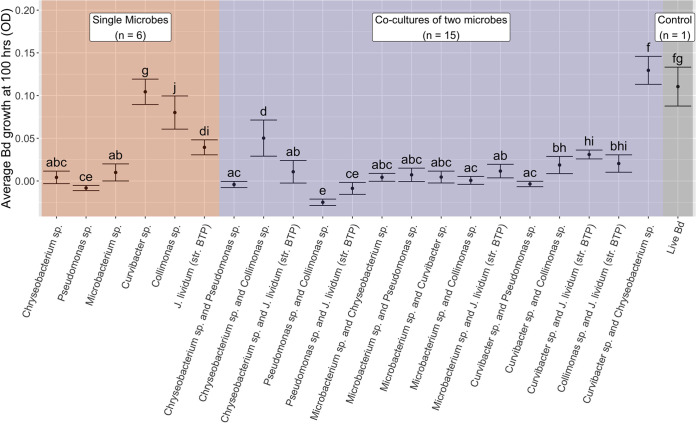
Assay results from the effect of CFS on *Bd* growth from bacteria that were grown together, showing the inhibition of *Bd* growth between single strain and multistrain CFS treatments (see panels A and B in Fig. 1). The average *Bd* growth at 100 h, measured as the optical density, in an experiment testing the effect of CFS on *Bd* growth from bacteria grown in single strain and multistrain cultures. We tested 6 single microbes and 15 combinations (background color) with a live *Bd*-only control (10 replicates each). The mean and standard error from an ANOVA with Tukey’s HSD test are shown here with significance groupings (letters) above each error bar (ANOVA *P* value = 2.00 × 10^−16^; the Tukey’s HSD results are presented in Fig. S4 in the supplemental material).

Pseudomonas *LC2F* with *Collimonas TK10* and *Curvibacter CW54D* with *Collimonas TK10* were the only multistrain combinations that were better at inhibiting *Bd* than was either alone (one-way ANOVA, *P* = 2.00 × 10^−16^) (Tables S3 and S5; Fig. 2; Fig. S1G and J). There were seven treatments where one single-strain CFS was equal in *Bd* growth to its respective multistrain trial but the other single-strain CFS inhibited *Bd* less or not at all (e.g., Fig. S1H). Three treatments only had one single-strain CFS that was clearly better at inhibiting than the other or the multistrain combination (e.g., Fig. S1B). Overall, Pseudomonas *LC2F* CFS inhibited *Bd* the most, and Pseudomonas *LC2F*, *Chryseobacterium CW20D*, and *Microbacterium CW37E* were all more inhibitive than were the Burkholderiales order species (*Curvibacter CW54D*, *Collimonas TK10*, J. lividum [*str. BTP*]) (Fig. 2).

### Bacterial isolates grown together and separately.

**FIG 3:**
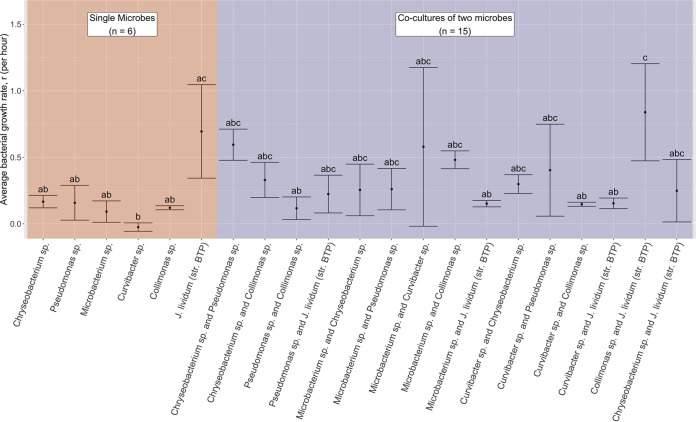
Assay results from bacterial isolates grown together and separately (see panel C in Fig. 1). The average bacterial growth rate (r) per hour, measured over 5 days, as estimated via growth curve modeling using optical density data, when bacteria were grown together or alone in broth tubes. We tested 6 single microbes and 15 combinations (background color). The mean and standard error from an ANOVA with Tukey’s HSD test are shown here with significance groupings (letters) above each error bar (ANOVA *P* value = 0.000603; the Tukey’s HSD results are presented in Table S4 in the supplemental material).

We compared the isolates’ abilities to grow together and alone using the carrying capacity (k) and the growth rate (r). Carrying capacity simply refers to a culture’s maximum possible growth, meaning the point at which the population growth reaches an asymptote such that cell death and birth rates equilibrate. The carrying capacity (k) was not significantly different across treatments (one-way ANOVA, *P* = 0.289) (Fig. S2). The growth rates (r) of different microbes were significantly different among the treatments (one-way ANOVA, *P* = 0.000603) (Table S4). *Curvibacter CW54D* was the slowest growing, whereas J. lividum (*str. BTP*) was the fastest growing, but it had the greatest variation (Fig. 3). Overall, most multistrain trials grew slightly faster than did their respective single-strain counterparts (Fig. 3).

### Separate-then-combined CFS assay.

**FIG 4:**
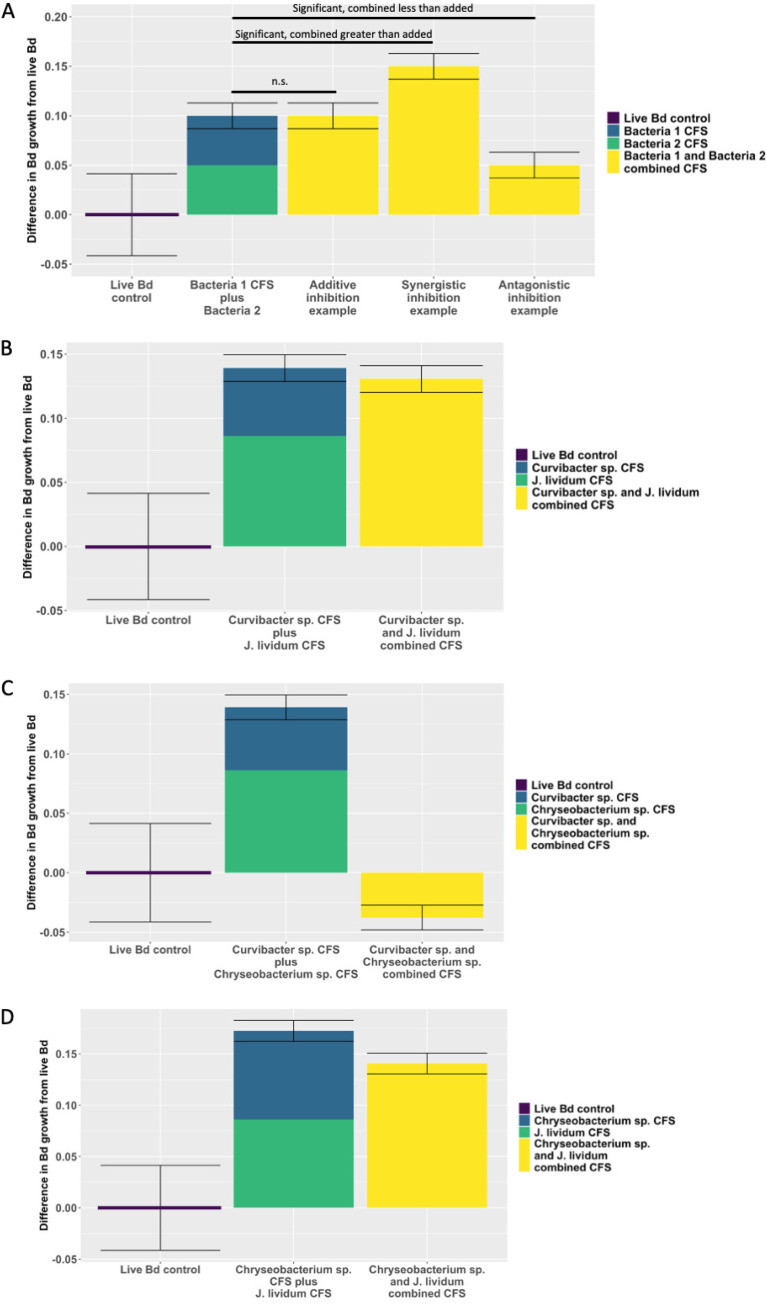
Separate-then-combined CFS assay results showing the inhibition of *Bd* growth between single strain and multistrain CFS treatments. The difference in *Bd* growth at 100 h when grown with bacterial CFS, compared to the live *Bd* control, measured as the optical density, in an experiment testing the separate-then-combined CFS effect on *Bd* growth (calculated as the live *Bd* OD minus the CFS treatment OD). (A) A model example of what the graph would look like for additive, synergistic, and antagonistic results. (B) An example of the only additive interaction that we found, which was between *Curvibacter CW54D* and J. lividum (*str. BTP*) (*t* test, *t* statistic = 0.953, *P* value = 0.372, mean of differences = 0.00827). (C) An example of a highly antagonistic interaction between *Curvibacter CW54D* and *Chryseobacterium CW20D* (*t* test, *t* statistic = 29.3, *P* value = 1.98 × 10^−9^, mean of differences = 0.232). (D) An example of the majority of the antagonistic interactions that we observed, wherein the addition of the individual metabolite effects of *Chryseobacterium CW20D* and J. lividum (*str. BTP*) were higher than the metabolites produced when they were grown together (as in panel C). However, the overall difference in *Bd* growth between the paired and individual trials was not significantly different on its own; however, even if it were significant, it had a small difference in means (*t* test, *t* statistic = 9.93, *P* value = 2.24 × 10^−5^, mean of differences = 0.0869). Color denotes whether the CFS was derived from one bacterial strain, derived from a two-strain combination, or included no CFS (in the case of the live *Bd* control). The mean and standard error from a *t* test comparing the sum of the individual CFS treatments with the combined CFS treatment are shown in the bar. The full results from all comparisons can be found in Table S7 in the supplemental material.

We found no synergistic interactions and one additive interaction (between *Curvibacter CW54D* and J. lividum [*str. BTP*]) (*t* test, *P* = 0.9533; meaning that the sum of the single-strains was not significantly different than their combination and was thus additive) (Tables S6 and S7; Fig. 4B). All other comparisons were antagonistic (e.g., Fig. 4C and D; Fig. S3; Tables S6 and 7). Of these, we observed a few different outcomes: (i) no difference between single strain and multistrain CFS; (ii) multistrain CFS and one single-strain CFS were the same, and other single-strains had more or less *Bd* inhibition than did the former; and (iii) the multistrain CFS was far less effective than either individual isolate (Fig. S3; Table S6). This last case of highly antagonistic interaction occurred in *Chryseobacterium CW20D* with either *Curvibacter CW54D* or *Collimonas TK10* (Fig. 4C; Fig. S3B and C; Tables S6 and 7).

## DISCUSSION

Although J. lividum (*str. BTP*) strains have been successful at inhibiting *Bd* in some amphibians during some *in vitro* and *in vivo* experimental trials (12, 13, 26), they have not always shown consistent results in all amphibians, and few efforts have tested alternative strains or multistrain probiotics in the host described here (32, 33, 36, 37, 56, 58). Boreal toads (*Anaxyrus boreas boreas*) in the montane systems of Colorado have been declining due to the spread of *Bd* in recent decades (41, 43), and wildlife managers are eager to discover more approaches that can mitigate *Bd*-related population declines. Experimental trials in the lab have demonstrated that inoculation with J. lividum (*str. BTP*) can increase survival by 40% when toads are challenged with *Bd* (12), but perhaps this figure could be improved by developing combinations of probiotics or by optimizing a new probiotic strain. Thus, this present study aimed to compare the activity of a variety of bacterial symbionts that were isolated from boreal toads *in vitro*, whether they might perform better than J. lividum (*str. BTP*), or whether cocktail combinations of bacteria could perform better against *Bd*. From a microbial ecology perspective, we also aimed to understand how the common bacterial skin symbionts of an amphibian may engage in additive, synergistic, or antagonistic interactions that can influence their capacity to inhibit the pathogen.

### Question one: are there other toad skin microbes that inhibit Bd, besides J. lividum (str. BTP)?

We found a number of *Bd* inhibitive isolates on boreal toad skin. Successful probiotics should also fulfill a few other important requirements: (i) be native to the system of study, so as to avoid the spread of nonnative organisms; (ii) be culturable and tractable for lab and field use; and (iii) not cause undesired effects to the host’s health or resident skin microbiota when isolate is applied to host skin (9, 10). Our data can only be used to directly evaluate the first two of these, given the limitations of *in vitro* studies, but our results indicate that the third point is an important consideration when creating probiotics. Related to the first point, we specifically isolated bacteria directly from the skins of wild boreal toads to ensure that the isolates were native to the system and would have a higher likelihood of colonizing and persisting on boreal toads. By the nature of *in vitro* experiments, point two is partially addressed, and the experimental isolates that we used were relatively fast-growing and were easy to manipulate in the lab. Further, isolates grew consistently over multiple passages in terms of the shape, color, and changes they made to the surrounding agar (e.g., Pseudomonas *LC2F* created a lime green color in the agar surrounding colonies, which we hypothesize are exudates that could be evaluated with untargeted metabolomics in the future). Finally, although we did not quantitatively test the final point, we found that certain interactions were antagonistic, and this might affect how probiotics establish and persist on a host.

Following the bacteria versus *Bd* lawn assays, we considered the culturability of the isolates and their tractability for probiotic use. Fast-growers, such as Pseudomonas *LC2F* and *Microbacterium CW37E*, had less contamination and allowed for faster preparation for field use. By comparison, J. lividum (*str. BTP*) had the most variable growth rate (Fig. 3). Depending on ambient lab factors, the growth rates on plates could sometimes be as slow as a week for Burkholderiales isolates and for J. lividum (*str. BTP*) in particular. We also noticed throughout our experiments that *Collimonas TK10* and J. lividum (*str. BTP*) cultures would lose the ability to produce violacein over consecutive plate transfers, whereas Pseudomonas *LC2F* and *Microbacterium CW37E* consistently produced a yellowish-green product within less than 24 h of growth. Overall, Pseudomonas *LC2F* was the best *Bd* inhibitor, and Pseudomonas *LC2F*, *Chryseobacterium CW20D*, and *Microbacterium CW37E* (which are all in different orders) were more inhibitive and tractable to culture than were any of the Burkholderiales taxa (Fig. 2). Burkholderiales isolates (*Curvibacter CW54D*, *Collimonas TK10*, and J. lividum [*str. BTP*]) were especially important for the comparisons here because these are highly abundant on many amphibians and include anti-*Bd*, violacein-producing isolates, which are often used against *Bd* in other amphibians, including boreal toads (13, 27–29, 34, 35, 55). In terms of how tractable this is in the context of producing probiotics, though, it does mean that the violacein producers that we studied were less consistent in culture than were the other isolates used in this study, in addition to being less *Bd*-inhibitive.

### Question two: are pairs of microbes more inhibitive than single microbe treatments?

Some combinations of isolates have been shown to be more effective than single-strain probiotics in other amphibians (36–40). In this study, *Curvibacter CW54D* or Pseudomonas *LC2F* with *Collimonas TK10* were more inhibitive than either respective single-strain CFS (Fig. 2). Further, to optimize the efficacy of violacein-producing bacteria, combining Pseudomonas *LC2F* or *Curvibacter CW54D* with *Collimonas TK10* or combining *Chryseobacterium CW20D* with J. lividum (*str. BTP*) would provide better inhibition than J. lividum (*str. BTP*) alone (Fig. 2). These combinations were more effective together than alone, which provides an important alternative set of probiotics to test in Colorado boreal toads.

A critical next step in this work is that we do not know how toad health is affected with a higher abundance of most of the *in vitro*-tested isolates or cocktails or their metabolites. Characterizing their metabolite profiles when confronted with *Bd* could shed some light on the safety of chemical compounds. Further, measuring the toad skin mucosome’s ability to defend against *Bd in vivo* pre-amendment and post-amendment is a useful way to test the effectiveness and safety of single strain and multistrain probiotics in a controlled manner, while also taking into account the existing microbiota on the host (24).

### Question three: how do *Bd*-inhibiting bacteria interact with each other?

We tested whether a probiotic cocktail mixture of bacteria inhibited *Bd* via facilitative means (which can include, but are not exclusively indicative of, exuded metabolites) and whether chemical metabolites were involved in these interactions (additive, synergistic, or antagonistic). Additionally, considering the pretreatment skin microbiome of a host is important so as to avoid unwanted interactions between probiotics and the existing microbes. It is reasonable to assume that this priority effect could cause probiotics to have trouble establishing or persisting, or it could result in varied success, depending on the condition of the prior host microbiota (49, 51). In this study, most of the metabolite interactions that we observed fit an antagonistic model (Fig. 4B and C; Fig. S3). We observed that *Curvibacter CW54D* and *Collimonas TK10* caused *Chryseobacterium CW20D* to decrease its inhibition due to strong antagonistic metabolite effects (although it is highly inhibitive on its own, otherwise).

We were particularly interested in how J. lividum (*str. BTP*) and other Burkholderiales bacteria interact, since isolates in this order are abundant on boreal toads and often have similar metabolic functions, so one might hypothesize that they compete for niche space on toad skin (27–29, 34, 35, 55). Based on our results, if J. lividum (*str. BTP*) continues being developed as a wild boreal toad probiotic, as it currently is, it is worthwhile to test the efficacy of *Curvibacter CW54D* applied to toad skin alongside J. lividum (*str. BTP*), as they inhibited *Bd* additively and grew at a higher rate when they were combined than did either alone (Table 2; Fig. 2 and 4A; Fig. S3). This is somewhat contrary to what we hypothesized, which is that existing Burkholderiales isolates might compete for niche space, and, thus, J. lividum might have trouble establishing and persisting if amended to toad skin during the late tadpole stage, when Burkholderiales order microbes are high (34, 35). It is possible that through competition, however, these two isolates are producing more metabolites and that these happen to inhibit *Bd* better than when either is growing alone.

**TABLE 2:** Possible interactions for each microbial pair, based on the results from the three mechanistic assays (CFS effect on *Bd* growth from bacteria grown together, separate-then-combined CFS, and bacterial isolates grown together and separately)^*a*^

Microbial pair	Summary of interactions	Outcome
Pseudomonas *LC2F* and *Microbacterium CW37E*	No enhanced growth rate, facilitative effect, or metabolite effect when grown together	Pseudomonas *LC2F* displayed only slightly stronger *Bd* inhibition than did *Microbacterium CW37E*, and there was no measured interaction between the two
Pseudomonas *LC2F* and *Curvibacter CW54D*	No enhanced growth rate, facilitative effect, or metabolite effect when grown together	*Curvibacter CW54D* did not affect the ability of Pseudomonas *LC2F* to inhibit *Bd*
Pseudomonas *LC2F* and *Collimonas TK10*	No enhanced growth rate, facilitative effect, or metabolite effect when grown together	Pseudomonas *LC2F* displayed stronger *Bd* inhibition than did *Collimonas TK10*, and there was no measured interaction between the two
Pseudomonas *LC2F* and J. lividum (*str. BTP*)	No enhanced growth rate, facilitative effect, or metabolite effect when grown together	Pseudomonas *LC2F* displayed stronger *Bd* inhibition than did J. lividum (*str. BTP*), and there was no measured interaction between the two
*Chryseobacterium CW20D* and Pseudomonas *LC2F*	Enhanced each other’s growth rate but did not have a metabolite or facilitative effect on *Bd*	These microbes may be interacting with each other, but this interaction does not increase *Bd* inhibition in any way
*Chryseobacterium CW20D* and *Microbacterium CW37E*	No enhanced growth rate, facilitative effect, or metabolite effect when grown together	These microbes did not change each other’s' ability to inhibit *Bd* when grown together
*Chryseobacterium CW20D* and *Curvibacter CW54D*	Enhanced each other’s' growth rate and increased *Bd* growth when their metabolites were together; the metabolite effect of *Chryseobacterium CW20D* on *Bd* disappeared when grown with Burkholderiales	*Curvibacter CW54D* may be competing with *Chryseobacterium CW20D* for space or resources; *Chryseobacterium CW20D* no longer puts resources into producing anti-*Bd* metabolite(s), which enhances *Bd* growth by some unknown mechanism
*Chryseobacterium CW20D* and *Collimonas TK10*	Enhanced each other’s growth rate but did not have a metabolite or facilitative effect on *Bd*	These microbes may be interacting with each other, but this interaction does not increase *Bd* inhibition in any way
*Chryseobacterium CW20D* and J. lividum (*str. BTP*)	No enhanced growth rate, facilitative effect, or metabolite effect when grown together	*Chryseobacterium CW20D* displayed stronger *Bd* inhibition than did J. lividum (*str. BTP*), and there was no measured interaction between the two
*Curvibacter CW54D* and *Microbacterium CW37E*	No enhanced growth rate, facilitative effect, or metabolite effect when grown together	*Curvibacter CW54D* did not affect the ability of *Microbacterium CW37E* to inhibit *Bd*
*Curvibacter CW54D* and *Collimonas TK10*	*Collimonas TK10* likely outgrew *Curvibacter CW54D* when grown together; a facilitative interaction was observed	These two microbes may induce a metabolite response from each other through competition, which subsequently inhibits *Bd*
*Curvibacter CW54D* and J. lividum (*str. BTP*)	J. lividum (*str. BTP*) grew less rapidly when grown with *Curvibacter CW54D*, and they exhibited an additive metabolite response	These two microbes may induce a metabolite response from each other through competition, which subsequently inhibits *Bd*
*Collimonas TK10* and *Microbacterium CW37E*	No enhanced growth rate, facilitative effect, or metabolite effect when grown together	*Microbacterium CW37E* displayed stronger *Bd* inhibition than did *Collimonas TK10*, and there was no measured interaction between the two
*Collimonas TK10* and J. lividum (*str. BTP*)	No enhanced growth rate, facilitative effect, or metabolite effect when grown together	J. lividum (*str. BTP*) displayed stronger *Bd* inhibition than did *Collimonas TK10*, and there was no measured interaction between the two
J. lividum (*str. BTP*) and *Microbacterium CW37E*	J. lividum (*str. BTP*) grew more rapidly alone than with *Microbacterium CW37E*, but there was no enhanced metabolic effect; *Microbacterium CW37E* inhibited *Bd* better than did J. lividum (*str. BTP*)	*Microbacterium CW37E* may be competing with J. lividum (*str. BTP*) for space or resources and inhibits *Bd* better

a The summary of interactions is based primarily on Fig. S1, S2, and S3.

In addition, there are a variety of ways in which multistrains interact and thereby inhibit *Bd* (Table 2). One example is *Collimonas TK10* and *Curvibacter CW54D.* This combination inhibits *Bd* especially well when the two microbes are grown together versus individually (Fig. 2; Fig. S1), however, it is not because of metabolite-induced effects, which are antagonistic (Fig. S3). It is also not the case that these microbes help each other grow faster; in fact, their combined growth rate is lower than the sum of each individual growth rate (Fig. 3). Thus, growth competition between *Collimonas TK10* and *Curvibacter CW54D* could be inducing increased metabolite production that consequently inhibits *Bd* well. Table 2 shows a summary of the possible outcomes that our results support for each combination of strains. In summary, we found that two-strain probiotic cocktails varied greatly in their mechanisms of action and that there was no one “typical” interaction between successful inhibitors of *Bd*. This means that mixed probiotics can potentially interact in unpredictable ways, which is an important consideration when designing future probiotic cocktails for further assessment. This variety in mechanism and interaction types could be one reason for the wide variation in how some hosts respond to probiotic treatment (49).

### Conclusion.

This study found that some combinations of microbes worked more effectively than did their individual counterparts and that probiotic cocktails inhibited *Bd* using a variety of different mechanisms. This finding suggests that the existing innate microbiome of individuals could affect how a probiotic establishes or persists and that multistrain combinations of microbes can act in unpredictable ways and should be tested on a case-by-case basis before use on a host. Further research into the stability of these interactions under different conditions is important, especially given the varied habitats in which amphibians live and seasonal or climatic changes over time. The scope of this study did not include identifying metabolites, but past studies suggest that it is an important next step. Our study also did not focus on the host safety of these isolates as probiotics, but this can be a goal of future studies. Such considerations are not only important in amphibians, but also in other wildlife in which traditional antibiotic therapies against widespread disease are either not feasible or in those in which there is a fear of pathogens developing resistance (5, 8).

## MATERIALS AND METHODS

Our workflow involved several steps, which are briefly described here and are elaborated below. These steps were designed to narrow down the targets for probiotic cocktail tests and are summarized in Fig. 1. We first isolated and identified bacteria directly from boreal toad skin from wild populations in Colorado. Then, we chose representatives from each distinct taxon to test against a lawn of *Bd* for inhibition. We cross-referenced candidate taxa with potential keystone members identified from a separate co-occurrence analysis on 16S amplicon read data so as to reduce our candidate taxa list to six bacterial isolates. To further characterize the mechanisms of inhibition in the five isolates, we conducted three types of assays: (i) an assay of one aspect of facilitation testing the effect of the cell-free supernatant (CFS) on *Bd* growth from bacteria grown together; (ii) an assay of another aspect of facilitation in which bacterial isolates were grown together and separately; and (iii) a separate-then-combined CFS assay to test the additive, synergistic, and antagonistic effects between metabolites (Fig. 1). Despite the limitations and challenges that come with *in vitro* studies, CFS assays are considered to be the most effective and consistent means by which to evaluate *Bd*-inhibition effects in a large number of cultured isolates (36, 37, 58). Together, these assays allow us to distinguish between three possible ways that mixed species probiotics may reduce *Bd* growth: via combining different metabolites from multiple isolates, improving the growth of isolates as a result of coculture, or increasing the metabolite production by isolates as a result of coculture.

Detailed protocols for each of the following steps and Sanger sequences that were used can be found on figshare (59, 60), and all of the R scripts can be found on GitHub at https://github.com/aalexiev/ProbioticCocktailsRepo.

### Isolation and identification of bacteria.

Bacteria were isolated over the course of two sampling years, namely, 2018 and 2019, from swabs of wild boreal toad skin in three wild sites in Chaffee County and Larimer County, Colorado, as well as captive, reared boreal toads in the Native Aquatic Species Restoration Facility (NASRF, run by Colorado Parks and Wildlife). The swab sampling of the toads was conducted with an approved IACUC protocol from the University of Colorado Boulder (protocol 2629) and a State of Colorado scientific collection permit (18HP0998, 19HP0998). All of the toads were rinsed with sterile DI water prior to having their entire body surfaces swabbed with a sterile rayon-tipped swab (BD BBL, East Rutherford, New Jersey). The swabs were then rubbed onto either R2A media or 1% tryptone media. Visually distinct bacteria were further isolated on their respective starting media until the colonies were pure.

We extracted DNA from pure cultures with a Qiagen DNeasy UltraClean Microbial Kit (Hilden, Germany), and it was then identified via the PCR amplification of the 16S rRNA region (using 27F– 1492R primers) and Sanger sequencing through Genewiz LLC (South Plainfield, NJ). We matched sequences to the NCBI 16S rRNA bacteria database, which is a curated nucleotide database of several resources, including GenBank, RefSeq, TPA, and PDB (61). This yielded 125 unique taxonomic groups (at either the species or genus level), from which one representative was chosen for downstream assays. Table 3 shows a summary of the origin of the isolates that were sequenced.

**TABLE 3:** The number of genetically unique bacterial isolates that originated from each location or were collected from each life stage, with NA meaning that the life stage was not captured or sampled at that location

Isolate	NASRF	Chaffee County site 1	Larimer County site 1	Larimer County site 2	Total per life stage
Adult (28 individuals)	8	10	6	57	81
Subadult (5 individuals)	NA	9	NA	NA	9
Metamorph (2 individuals)	NA	13	NA	NA	13
Tadpole (6 individuals)	NA	10	NA	1	11
Egg (4 masses)	NA	6	5	NA	11
Total per site	8	48	11	58	125

The only exceptions to the above protocol were J. lividum (*str. BTP*) and *Collimonas TK10*, and we already knew these isolate identities from previous sequencing efforts. Janthinobacterium lividum (*str. BTP*) was previously identified using a qPCR protocol using J. lividum primers, and it has been used in previously peer-reviewed and published *in vivo* studies (12) as well as in yet-unpublished and ongoing field studies. *Collimonas TK10* was determined via the short-read 16S marker gene sequencing of a pure culture colony as part of a separate, yet-unpublished, and ongoing project. The 16S short read was matched to the Silva database at 100% sequence identity. The *Collimonas TK10* sequence is included with the deposited Sanger sequences (54).

Further, we wanted to make sure that, for the duration of our experiments, we grew all of the isolates so that the culture was in the middle of its exponential growth phase. This would ensure that this parameter is held constant and that if a bacterial isolate failed to inhibit *Bd*, it would not be because it was in a lag or stationary phase. Thus, we measured the growth curve of each taxonomic representative via absorbance (OD_600_) in 96-well plates and used the growthcurver package in R (version 4.1.1) to estimate the exponential phase (62).

### Bacteria versus *Bd* lawn assays.

We tested each bacterial isolate against *Bd* on plates. The *Bd* strain JEL423 was obtained from collaborators in the form of frozen cryostocks, and it has been used in the past in other studies (57, 63, 64). To revive them, the stocks were thawed at room temperature, transferred to 1 to 2 mL fresh 1% tryptone media in a 50 mL flask, and grown at room temperature. Four-day-old *Bd* cultures from a flask were transferred to 1% tryptone agar plates and grown at room temperature for 5 to 7 days. *Bd* zoospores were harvested via plate washes. Plate washes averaging 1.7× 10^6^ zoospores/mL produced a lawn of *Bd* on 1% tryptone agar plates. Once dry, we spotted 2 μL of bacterial liquid culture onto the lawn, and we recorded photographs and measured the zone of inhibition on the plates every 24 h for at least 3 days.

### Selecting bacterial isolates for cocktails.

Due to the large number of isolates that had some measure of inhibition on the *Bd* lawn plates, we pared down the list of targets for further study. To do this, we took into consideration the amount of *Bd* inhibition from the *Bd* lawn assays (above), a co-occurrence analysis, and past literature.

A co-occurrence analysis identified bacteria that naturally co-occur frequently on wild boreal toads as potential multistrain probiotics that we could test in subsequent steps. We used BLAST to match the Sanger sequences of our bacterial isolates to a custom database of bacterial 16S data from wild toads at South Cottonwood (sequenced in 2019 as part of a separate, ongoing project) (56). The larger 16S data set was then filtered to show only matches to our Sanger sequenced bacteria that inhibited *Bd* lawn growth. We applied a centered log-ratio transformation to statistically account for the compositionality of the data, and then we calculated the Spearman coefficients between isolates (ρ > 0.5, *P* < 0.01) (Table S1).

The above criteria, along with information from past literature, led us to choose the following isolates to test in multistrain inhibition assays: Pseudomonas *LC2F*, *Chryseobacterium CW20D*, *Microbacterium CW37F*, *Collimonas TK10*, J. lividum (*str. BTP*), and *Curvibacter CW54D.*

### CFS effect on *Bd* growth from bacteria grown together.

We considered whether the increased *Bd*-inhibition was due to bacterial partners facilitating each other’s *Bd* inhibition. This relates to interactions (a) and (b) in the facilitation model shown in Fig. 1. To assess the facilitation, bacteria were grown together for 4 days prior to having their CFS harvested. We combined two isolates during their respective exponential growth phases.

We had several types of controls: (i) each bacterial isolate from which to harvest CFS, grown individually; (2) live *Bd* zoospores (6.5× 10^5^ zoospores/mL); (3) each CFS (both single and multistrain) with no *Bd* added; and (4) sterile media for comparison. Each control and trial were replicated 10 times. Once the bacterial CFS was harvested, we added *Bd* zoospores (6.5× 10^5^ zoospores/mL) to each individual and combined CFS. The plates grew at room temperature as we measured the OD_600_ value approximately every 24 h for 5 days.

### Bacterial isolates grown together and separately.

As part of assessing facilitation interaction (c) in the model shown in Fig. 1, we tested whether cocultures of bacteria resulted in greater growth, compared to monocultures of bacteria. We grew all of the isolates in pairs and alone in 1% tryptone broth at room temperature to their respective exponential growth phases (ranging from 24 to 48 h). We then measured their growth (OD_600_) every 12 h for 2 days, at which point the isolates would have reached the end of their exponential growth phases. We wanted to ensure that the OD counts were representative of mostly live bacteria by the end of the experiment (as opposed to dead cells), so we also plated a dilution series of the cultures on the fifth day and counted live colonies 24 to 48 h later (depending on when the colonies grew enough to be countable). Unfortunately, because most of the bacterial isolates looked similar and could conceivably change morphology or color when grown in pairs, we could not accurately count the separate species in coculture on the plates. However, when possible, we noted that the isolates grew in approximately equal amounts on the plates.

### Separate-then-combined CFS assay.

We asked whether combining metabolites from different isolates resulted in greater *Bd* inhibition, compared to metabolites from one isolate alone (Fig. 1). We grew bacteria in 1% tryptone broth for 4 days at room temperature to ensure that the medium was spent (based on earlier growth curves). Once grown, the bacterial cultures were syringe-filtered through a sterile 0.22 μm filter, which created a cell-free supernatant (CFS). The CFS was then prepared for single versus combined trials in 96-well plates in two ways: (i) 50 μL of CFS from a single strain was inoculated directly with 50 μL of *Bd* zoospores (4.0× 10^6^ zoospores/mL); and (ii) 25 μL of each of two strains were combined, resulting in a total of 50 μL in the well, and inoculated with 50 μL *Bd*. To standardize the OD measurements across treatments, we also measured the OD of each CFS without *Bd* added and subtracted this from each CFS treatment. Our positive control was live *Bd* zoospores in 1% tryptone, standardized against sterile media. We prepared 10 replicates of each treatment and control. The plates grew at room temperature, and we measured the OD_600_ values approximately every 24 h for 5 days.

### Statistical analysis of mechanistic assays.

We used the OD data from the two CFS-based experiments and from the bacterial isolates grown together and separately. We used the growthcurver package to fit a growth curve to the OD data and identify outliers. The growth curve was fit to a standard logistic equation with parameters for the growth rate (r), the initial population size, and the carrying capacity (k). Outliers are identified as those with unusually large sigma values (and thus being poor fits). Here, the sigma values are the residual sums of squares from the fit of the logistic curve to the OD data. The fitted curves and outlier choices were each checked visually on plots of the OD data with the fitted curve to ensure that they made logical sense, given the parameters of the assays. Then, we filtered the outliers and subtracted the OD values for the sterile media from each remaining sample to normalize the OD values. These filtered and normalized OD values were then used for the calculations related to the specific assay and the question to which each assay was related.

When analyzing the CFS effect on *Bd* growth from bacteria that were grown together, we kept the OD as the mean *Bd* growth of each trial at 100 h for ease of visualization. Then, we performed a one-way ANOVA with a Tukey-Kramer *post hoc* analysis on each multistrain pair and on its corresponding single-strain trials independently, which helped us determine whether a multistrain pair was more or less inhibitive than its corresponding single-strain components (e.g., only comparing *Chryseobacterium CW20D*, J. lividum (*str. BTP*), and *Chryseobacterium CW20D* plus J. lividum [*str. BTP*]). We also performed a one-way ANOVA with a Tukey-Kramer *post hoc* analysis to determine which CFS trials (single strain or multistrain) had the overall highest *Bd* inhibition across all of the strains and combinations tested.

Relating to the OD data from the bacterial isolates grown together and separately, we used the growth rate (r) and carrying capacity (k) for the bacteria from the fitted logistic curve from growthcurver. We used these parameters because the OD was taken over time, and our question related to this assay was whether bacteria induce each other to grow faster or more (hence, the use of the growth rate and carrying capacity).Then, we calculated two sets of statistics for each of the r and k values, and we created representative graphs of: (i) a one-way ANOVA with a Tukey-Kramer *post hoc* analysis on each multistrain pair and its corresponding single-strain trials and (ii) a one-way ANOVA with Tukey-Kramer *post hoc* analysis on the whole data set of all of the single strain and multistrain CFS with *Bd*. The former represents whether a multistrain pair is more or less inhibitive than its corresponding single-strain components (e.g., only comparing *Chryseobacterium CW20D*, J. lividum [*str. BTP*], and *Chryseobaterium CW20D* plus J. lividum [*str. BTP*]), whereas the latter represents how trials compare (single strain or multistrain) across all of the strains that were used and which is the most or least inhibitive overall, in the entire experiment.

For the separate-then-combined CFS assay, we calculated the difference of the OD values between the mean of the positive *Bd* controls and that of each trial (single strain and multistrain CFS with *Bd*). This gave us the amount of *Bd* inhibition of each trial, which is the main measure of additive, synergistic, or antagonistic function. Further, to determine which samples were additive, synergistic, or antagonistic in the separate-then-combined CFS assay, we tested whether the sum of the *Bd* inhibition of the single-strain CFS treatments was significantly different than that of the corresponding multistrain CFS. We then used these results to evaluate which trials were additive, synergistic, or antagonistic, wherein: (i) additive inhibition occurred when the inhibition caused by two sets of metabolites equaled their sum when they are combined (*t* test results were not statistically significant); (ii) synergistic inhibition occurred when the inhibition caused by two sets of metabolites was more than their sum when combined (*t* test results were statistically significant, and the *t* test mean of the differences was less than zero); and (iii) antagonistic inhibition occurred when the inhibition of two sets of metabolites was less than their sum when combined (*t* test results were statistically significant, and the *t* test mean of the differences was greater than zero).

All of the statistical analyses were done in R (version 4.0.5 [2021-03-31]), and the published graphs were made with ggplot2.

### Data availability.

All data and sequences are on figshare (60, 65), and the scripts are on GitHub (https://github.com/aalexiev/ProbioticCocktailsRepo).
