# Supplementary material for: Together or Alone: Evaluating the Pathogen Inhibition Potential of Bacterial Cocktails against an Amphibian Pathogen
Source: Microbiol Spectr. 2023 Jan 31;11(2):e01518-22. doi: 10.1128/spectrum.01518-22 (PMC10100949; doi:10.1128/spectrum.01518-22)
Supplement: Supplemental file 1 — Supplemental material. Download spectrum.01518-22-s0001.pdf, PDF file, 2.0 MB [file spectrum.01518-22-s0001.pdf]

Supplementary Table 1: Sequence identity match information is provided for four of the bacterial isolates used in the mechanistic assays. The other two bacterial isolates used were already previously identified for independent projects using a different set of methods. Taxonomy match parameters were obtained from BLAST. Many of these isolates matched with an existing cultured strain identified to species level, however, since some had percent identity matches less than 100 and some gaps, we chose to only use genus level identifications in this manuscript.

Isolate name used in manuscript	Percent identity match (%)	Alignment length (bp)	Number mismatches (bp)	Number gap openings (bp)	E value	Bitscore	Accession number of NCBI match	Taxonomic name of NCBI match
Chryseobacterium CW20F	98.65	813	4	7	0	1441	NR_042642	Chryseobacterium aquaticum strain 10-46 16S ribosomal RNA, partial sequence
Microbacterium CW37E	95.41	828	23	14	0	1323	NR_114984	Microbacterium chocolatum strain DSM 12507 16S ribosomal RNA, partial sequence
Pseudomonas LC2F	98.35	787	6	6	0	1382	NR_024909	Pseudomonas orientalis strain CFML 96-170 16S ribosomal RNA, partial sequence
Curvibacter CW54D	96.92	812	24	1	0	1376	NR_113696	Curvibacter delicatus strain NBRC 14919 16S ribosomal RNA, partial sequence

Supplementary Table 2: Statistical results from co-occurrence analysis of OTUs in a 16S bacterial dataset taken from the skin of wild CO boreal toads. Spearman rho and p-values for correlations between pairs of OTUs are shown. This helped us decide which of the existing isolated bacteria, taken from the same ecosystem the 16S data was produced from, might be most likely to function facilitatively against *Bd*.

OTU	Correlated OTU	Spearman rho (only rho > 0.5 shown)	P-value (only significant listed, alpha = 0.01)
Bacteria;Proteobacteria;Gammaproteobacteria;Betaproteobacteriales;Burkholderiaceae;NA	Bacteria;Proteobacteria;Alphaproteobacteria;Acetobacterales;Acetobacteraceae;Belnapia	1	0
Bacteria;Proteobacteria;Gammaproteobacteria;Pseudomonadales;Pseudomonadaceae;Pseudomonas	Bacteria;Proteobacteria;Gammaproteobacteria;Pseudomonadales;Pseudomonadaceae;Pseudomonas	1	0
Bacteria;Proteobacteria;Gammaproteobacteria;Pseudomonadales;Pseudomonadaceae;Pseudomonas	Bacteria;Proteobacteria;Gammaproteobacteria;Pseudomonadales;Pseudomonadaceae;Pseudomonas	1	0
Bacteria;Proteobacteria;Gammaproteobacteria;Betaproteobacteriales;Burkholderiaceae;NA	Bacteria;Proteobacteria;Alphaproteobacteria;Rhizobiales;Beijerinckiaceae;Bosea	1	0
Bacteria;Proteobacteria;Gammaproteobacteria;Pseudomonadales;Pseudomonadaceae;Pseudomonas	Bacteria;Proteobacteria;Gammaproteobacteria;Pseudomonadales;Pseudomonadaceae;Pseudomonas	1	0
Bacteria;Proteobacteria;Gammaproteobacteria;Pseudomonadales;Pseudomonadaceae;Pseudomonas	Bacteria;Proteobacteria;Gammaproteobacteria;Pseudomonadales;Pseudomonadaceae;Pseudomonas	1	0
Bacteria;Proteobacteria;Gammaproteobacteria;Pseudomonadales;Pseudomonadaceae;Pseudomonas	Bacteria;Proteobacteria;Gammaproteobacteria;Pseudomonadales;Pseudomonadaceae;Pseudomonas	1	0
Bacteria;Proteobacteria;Gammaproteobacteria;Pseudomonadales;Pseudomonadaceae;Pseudomonas	Bacteria;Firmicutes;Bacilli;Bacillales;Family_XII;Exiguobacterium	1	0
Bacteria;Proteobacteria;Gammap	Bacteria;Firmicutes;Bacilli;Bacillal	1	0

roteobacteria;Pseudomonadales;Pseudomonadaceae;Pseudomonas	es;Family_XII;Exiguobacterium		
Bacteria;Proteobacteria;Gammaproteobacteria;Betaproteobacteriales;Burkholderiaceae;NA	Bacteria;Deinococcus-Thermus;Deinococci;Deinococcales;Deinococcaceae;Deinococcus	1	0
Bacteria;Proteobacteria;Gammaproteobacteria;Betaproteobacteriales;Burkholderiaceae;NA	Bacteria;Proteobacteria;Gammaproteobacteria;Pseudomonadales;Pseudomonadaceae;Pseudomonas	1	0
Bacteria;Bacteroidetes;Bacteroidia;Flavobacteriales;Weeksellaceae;Chryseobacterium	Bacteria;Proteobacteria;Alphaproteobacteria;Sphingomonadales;Sphingomonadaceae;Sphingomonas	1	0
Bacteria;Proteobacteria;Gammaproteobacteria;Pseudomonadales;Pseudomonadaceae;Pseudomonas	Bacteria;Proteobacteria;Gammaproteobacteria;Pseudomonadales;Pseudomonadaceae;Pseudomonas	1	0
Bacteria;Bacteroidetes;Bacteroidia;Flavobacteriales;Weeksellaceae;Chryseobacterium	Bacteria;Proteobacteria;Alphaproteobacteria;Sphingomonadales;Sphingomonadaceae;Sphingomonas	1	0

Supplementary Table 3: Tukey HSD results from ANOVA associated with Figure 2 in the main manuscript text.

Treatment Comparison	Difference in mean value	Lower 95% confidence interval	Upper 95% confidence interval	Adjusted p-value (Tukey HSD)
20D_Bd-20D_2F_Bd	0.0083	-0.0089713	0.02557132	9.79E-01
20D_Jliv10_Bd-20D_2F_Bd	0.0543	0.03702868	0.07157132	6.89E-14
20D_JlivBTP_Bd-20D_2F_Bd	0.0149	-0.0023713	0.03217132	1.98E-01
2F_Bd-20D_2F_Bd	-0.0041	-0.0213713	0.01317132	1.00E+00
2F_Jliv10_Bd-20D_2F_Bd	-0.0208333	-0.0385779	-0.0030887	5.51E-03
2F_JlivBTP_Bd-20D_2F_Bd	-0.0045	-0.0217713	0.01277132	1.00E+00
37E_20D_Bd-20D_2F_Bd	0.0084	-0.0088713	0.02567132	9.77E-01
37E_2F_Bd-20D_2F_Bd	0.0113	-0.0059713	0.02857132	7.16E-01
37E_54D_Bd-20D_2F_Bd	0.0087	-0.0085713	0.02597132	9.66E-01
37E_Bd-20D_2F_Bd	0.0141	-0.0031713	0.03137132	2.88E-01
37E_Jliv10_Bd-20D_2F_Bd	0.0048	-0.0124713	0.02207132	1.00E+00
37E_JlivBTP_Bd-20D_2F_Bd	0.0157	-0.0015713	0.03297132	1.30E-01
54D_20D_Bd-20D_2F_Bd	0.133625	0.115306	0.151944	9.21E-15
54D_2F_Bd-20D_2F_Bd	0.0006	-0.0166713	0.01787132	1.00E+00
54D_Bd-20D_2F_Bd	0.1085	0.09122868	0.12577132	9.21E-15
54D_Jliv10_Bd-20D_2F_Bd	0.0228	0.00552868	0.04007132	6.30E-04
54D_JlivBTP_Bd-20D_2F_Bd	0.0351	0.01782868	0.05237132	1.01E-09
Jliv10_Bd-20D_2F_Bd	0.08423333	0.06648874	0.10197793	9.21E-15
Jliv10_JlivBTP_Bd-20D_2F_Bd	0.024525	0.006206	0.042844	4.69E-04
JlivBTP_Bd-20D_2F_Bd	0.043475	0.025156	0.061794	7.86E-13

Live_Bd-20D_2F_Bd	0.1146	0.09175219	0.13744781	9.21E-15
20D_Jliv10_Bd-20D_Bd	0.046	0.02872868	0.06327132	2.08E-13
20D_JlivBTP_Bd-20D_Bd	0.0066	-0.0106713	0.02387132	9.99E-01
2F_Bd-20D_Bd	-0.0124	-0.0296713	0.00487132	5.41E-01
2F_Jliv10_Bd-20D_Bd	-0.0291333	-0.0468779	-0.0113887	2.45E-06
2F_JlivBTP_Bd-20D_Bd	-0.0128	-0.0300713	0.00447132	4.77E-01
37E_20D_Bd-20D_Bd	1.00E-04	-0.0171713	0.01737132	1.00E+00
37E_2F_Bd-20D_Bd	0.003	-0.0142713	0.02027132	1.00E+00
37E_54D_Bd-20D_Bd	0.0004	-0.0168713	0.01767132	1.00E+00
37E_Bd-20D_Bd	0.0058	-0.0114713	0.02307132	1.00E+00
37E_Jliv10_Bd-20D_Bd	-0.0035	-0.0207713	0.01377132	1.00E+00
37E_JlivBTP_Bd-20D_Bd	0.0074	-0.0098713	0.02467132	9.95E-01
54D_20D_Bd-20D_Bd	0.125325	0.107006	0.143644	9.21E-15
54D_2F_Bd-20D_Bd	-0.0077	-0.0249713	0.00957132	9.91E-01
54D_Bd-20D_Bd	0.1002	0.08292868	0.11747132	9.21E-15
54D_Jliv10_Bd-20D_Bd	0.0145	-0.0027713	0.03177132	2.40E-01
54D_JlivBTP_Bd-20D_Bd	0.0268	0.00952868	0.04407132	1.27E-05
Jliv10_Bd-20D_Bd	0.07593333	0.05818874	0.09367793	9.21E-15
Jliv10_JlivBTP_Bd-20D_Bd	0.016225	-0.002094	0.034544	1.61E-01
JlivBTP_Bd-20D_Bd	0.035175	0.016856	0.053494	1.04E-08
Live_Bd-20D_Bd	0.1063	0.08345219	0.12914781	9.21E-15
20D_JlivBTP_Bd-20D_Jliv10_Bd	-0.0394	-0.0566713	-0.0221287	4.66E-12
2F_Bd-20D_Jliv10_Bd	-0.0584	-0.0756713	-0.0411287	1.55E-14
2F_Jliv10_Bd-20D_Jliv10_Bd	-0.0751333	-0.0928779	-0.0573887	9.21E-15
2F_JlivBTP_Bd-20D_Jliv10_Bd	-0.0588	-0.0760713	-0.0415287	1.48E-14

37E_20D_Bd-20D_Jliv10_Bd	-0.0459	-0.0631713	-0.0286287	2.14E-13
37E_2F_Bd-20D_Jliv10_Bd	-0.043	-0.0602713	-0.0257287	2.58E-13
37E_54D_Bd-20D_Jliv10_Bd	-0.0456	-0.0628713	-0.0283287	2.19E-13
37E_Bd-20D_Jliv10_Bd	-0.0402	-0.0574713	-0.0229287	1.80E-12
37E_Jliv10_Bd-20D_Jliv10_Bd	-0.0495	-0.0667713	-0.0322287	2.00E-13
37E_JlivBTP_Bd-20D_Jliv10_Bd	-0.0386	-0.0558713	-0.0213287	1.27E-11
54D_20D_Bd-20D_Jliv10_Bd	0.079325	0.061006	0.097644	9.21E-15
54D_2F_Bd-20D_Jliv10_Bd	-0.0537	-0.0709713	-0.0364287	8.52E-14
54D_Bd-20D_Jliv10_Bd	0.0542	0.03692868	0.07147132	7.07E-14
54D_Jliv10_Bd-20D_Jliv10_Bd	-0.0315	-0.0487713	-0.0142287	7.31E-08
54D_JlivBTP_Bd-20D_Jliv10_Bd	-0.0192	-0.0364713	-0.0019287	1.28E-02
Jliv10_Bd-20D_Jliv10_Bd	0.02993333	0.01218874	0.04767793	1.05E-06
Jliv10_JlivBTP_Bd-20D_Jliv10_Bd	-0.029775	-0.048094	-0.011456	3.33E-06
JlivBTP_Bd-20D_Jliv10_Bd	-0.010825	-0.029144	0.007494	8.58E-01
Live_Bd-20D_Jliv10_Bd	0.0603	0.03745219	0.08314781	2.18E-13
2F_Bd-20D_JlivBTP_Bd	-0.019	-0.0362713	-0.0017287	1.49E-02
2F_Jliv10_Bd-20D_JlivBTP_Bd	-0.0357333	-0.0534779	-0.0179887	1.49E-09
2F_JlivBTP_Bd-20D_JlivBTP_Bd	-0.0194	-0.0366713	-0.0021287	1.10E-02
37E_20D_Bd-20D_JlivBTP_Bd	-0.0065	-0.0237713	0.01077132	9.99E-01
37E_2F_Bd-20D_JlivBTP_Bd	-0.0036	-0.0208713	0.01367132	1.00E+00
37E_54D_Bd-20D_JlivBTP_Bd	-0.0062	-0.0234713	0.01107132	1.00E+00
37E_Bd-20D_JlivBTP_Bd	-0.0008	-0.0180713	0.01647132	1.00E+00
37E_Jliv10_Bd-20D_JlivBTP_Bd	-0.0101	-0.0273713	0.00717132	8.69E-01

37E_JlivBTP_Bd-20D_JlivBTP_Bd	0.0008	-0.0164713	0.01807132	1.00E+00
54D_20D_Bd-20D_JlivBTP_Bd	0.118725	0.100406	0.137044	9.21E-15
54D_2F_Bd-20D_JlivBTP_Bd	-0.0143	-0.0315713	0.00297132	2.63E-01
54D_Bd-20D_JlivBTP_Bd	0.0936	0.07632868	0.11087132	9.21E-15
54D_Jliv10_Bd-20D_JlivBTP_Bd	0.0079	-0.0093713	0.02517132	9.88E-01
54D_JlivBTP_Bd-20D_JlivBTP_Bd	0.0202	0.00292868	0.03747132	5.86E-03
Jliv10_Bd-20D_JlivBTP_Bd	0.06933333	0.05158874	0.08707793	9.21E-15
Jliv10_JlivBTP_Bd-20D_JlivBTP_Bd	0.009625	-0.008694	0.027944	9.48E-01
JlivBTP_Bd-20D_JlivBTP_Bd	0.028575	0.010256	0.046894	1.10E-05
Live_Bd-20D_JlivBTP_Bd	0.0997	0.07685219	0.12254781	9.21E-15
2F_Jliv10_Bd-2F_Bd	-0.0167333	-0.0344779	0.00101126	9.25E-02
2F_JlivBTP_Bd-2F_Bd	-0.0004	-0.0176713	0.01687132	1.00E+00
37E_20D_Bd-2F_Bd	0.0125	-0.0047713	0.02977132	5.25E-01
37E_2F_Bd-2F_Bd	0.0154	-0.0018713	0.03267132	1.53E-01
37E_54D_Bd-2F_Bd	0.0128	-0.0044713	0.03007132	4.77E-01
37E_Bd-2F_Bd	0.0182	0.00092868	0.03547132	2.66E-02
37E_Jliv10_Bd-2F_Bd	0.0089	-0.0083713	0.02617132	9.57E-01
37E_JlivBTP_Bd-2F_Bd	0.0198	0.00252868	0.03707132	8.05E-03
54D_20D_Bd-2F_Bd	0.137725	0.119406	0.156044	9.21E-15
54D_2F_Bd-2F_Bd	0.0047	-0.0125713	0.02197132	1.00E+00
54D_Bd-2F_Bd	0.1126	0.09532868	0.12987132	9.21E-15
54D_Jliv10_Bd-2F_Bd	0.0269	0.00962868	0.04417132	1.15E-05
54D_JlivBTP_Bd-2F_Bd	0.0392	0.02192868	0.05647132	5.97E-12
Jliv10_Bd-2F_Bd	0.08833333	0.07058874	0.10607793	9.21E-15
Jliv10_JlivBTP_Bd-2F_Bd	0.028625	0.010306	0.046944	1.05E-05

JlivBTP_Bd-2F_Bd	0.047575	0.029256	0.065894	2.15E-13
Live_Bd-2F_Bd	0.1187	0.09585219	0.14154781	9.21E-15
2F_JlivBTP_Bd-2F_Jliv10_Bd	0.01633333	-0.0014113	0.03407793	1.16E-01
37E_20D_Bd-2F_Jliv10_Bd	0.02923333	0.01148874	0.04697793	2.21E-06
37E_2F_Bd-2F_Jliv10_Bd	0.03213333	0.01438874	0.04987793	9.46E-08
37E_54D_Bd-2F_Jliv10_Bd	0.02953333	0.01178874	0.04727793	1.61E-06
37E_Bd-2F_Jliv10_Bd	0.03493333	0.01718874	0.05267793	3.82E-09
37E_Jliv10_Bd-2F_Jliv10_Bd	0.02563333	0.00788874	0.04337793	8.25E-05
37E_JlivBTP_Bd-2F_Jliv10_Bd	0.03653333	0.01878874	0.05427793	5.72E-10
54D_20D_Bd-2F_Jliv10_Bd	0.15445833	0.13569246	0.17322421	9.21E-15
54D_2F_Bd-2F_Jliv10_Bd	0.02143333	0.00368874	0.03917793	3.41E-03
54D_Bd-2F_Jliv10_Bd	0.12933333	0.11158874	0.14707793	9.21E-15
54D_Jliv10_Bd-2F_Jliv10_Bd	0.04363333	0.02588874	0.06137793	2.92E-13
54D_JlivBTP_Bd-2F_Jliv10_Bd	0.05593333	0.03818874	0.07367793	7.04E-14
Jliv10_Bd-2F_Jliv10_Bd	0.10506667	0.0868611	0.12327224	9.21E-15
Jliv10_JlivBTP_Bd-2F_Jliv10_Bd	0.04535833	0.02659246	0.06412421	4.25E-13
JlivBTP_Bd-2F_Jliv10_Bd	0.06430833	0.04554246	0.08307421	1.25E-14
Live_Bd-2F_Jliv10_Bd	0.13543333	0.11222569	0.15864097	9.21E-15
37E_20D_Bd-2F_JlivBTP_Bd	0.0129	-0.0043713	0.03017132	4.61E-01
37E_2F_Bd-2F_JlivBTP_Bd	0.0158	-0.0014713	0.03307132	1.23E-01
37E_54D_Bd-2F_JlivBTP_Bd	0.0132	-0.0040713	0.03047132	4.14E-01
37E_Bd-2F_JlivBTP_Bd	0.0186	0.00132868	0.03587132	2.00E-02
37E_Jliv10_Bd-2F_JlivBTP_Bd	0.0093	-0.0079713	0.02657132	9.35E-01

37E_JlivBTP_Bd-2F_JlivBTP_Bd	0.0202	0.00292868	0.03747132	5.86E-03
54D_20D_Bd-2F_JlivBTP_Bd	0.138125	0.119806	0.156444	9.21E-15
54D_2F_Bd-2F_JlivBTP_Bd	0.0051	-0.0121713	0.02237132	1.00E+00
54D_Bd-2F_JlivBTP_Bd	0.113	0.09572868	0.13027132	9.21E-15
54D_Jliv10_Bd-2F_JlivBTP_Bd	0.0273	0.01002868	0.04457132	7.56E-06
54D_JlivBTP_Bd-2F_JlivBTP_Bd	0.0396	0.02232868	0.05687132	3.65E-12
Jliv10_Bd-2F_JlivBTP_Bd	0.08873333	0.07098874	0.10647793	9.21E-15
Jliv10_JlivBTP_Bd-2F_JlivBTP_Bd	0.029025	0.010706	0.047344	7.06E-06
JlivBTP_Bd-2F_JlivBTP_Bd	0.047975	0.029656	0.066294	2.22E-13
Live_Bd-2F_JlivBTP_Bd	0.1191	0.09625219	0.14194781	9.21E-15
37E_2F_Bd-37E_20D_Bd	0.0029	-0.0143713	0.02017132	1.00E+00
37E_54D_Bd-37E_20D_Bd	0.0003	-0.0169713	0.01757132	1.00E+00
37E_Bd-37E_20D_Bd	0.0057	-0.0115713	0.02297132	1.00E+00
37E_Jliv10_Bd-37E_20D_Bd	-0.0036	-0.0208713	0.01367132	1.00E+00
37E_JlivBTP_Bd-37E_20D_Bd	0.0073	-0.0099713	0.02457132	9.96E-01
54D_20D_Bd-37E_20D_Bd	0.125225	0.106906	0.143544	9.21E-15
54D_2F_Bd-37E_20D_Bd	-0.0078	-0.0250713	0.00947132	9.90E-01
54D_Bd-37E_20D_Bd	0.1001	0.08282868	0.11737132	9.21E-15
54D_Jliv10_Bd-37E_20D_Bd	0.0144	-0.0028713	0.03167132	2.52E-01
54D_JlivBTP_Bd-37E_20D_Bd	0.0267	0.00942868	0.04397132	1.41E-05
Jliv10_Bd-37E_20D_Bd	0.07583333	0.05808874	0.09357793	9.21E-15
Jliv10_JlivBTP_Bd-37E_20D_Bd	0.016125	-0.002194	0.034444	1.69E-01
JlivBTP_Bd-37E_20D_Bd	0.035075	0.016756	0.053394	1.16E-08

Live_Bd-37E_20D_Bd	0.1062	0.08335219	0.12904781	9.21E-15
37E_54D_Bd-37E_2F_Bd	-0.0026	-0.0198713	0.01467132	1.00E+00
37E_Bd-37E_2F_Bd	0.0028	-0.0144713	0.02007132	1.00E+00
37E_Jliv10_Bd-37E_2F_Bd	-0.0065	-0.0237713	0.01077132	9.99E-01
37E_JlivBTP_Bd-37E_2F_Bd	0.0044	-0.0128713	0.02167132	1.00E+00
54D_20D_Bd-37E_2F_Bd	0.122325	0.104006	0.140644	9.21E-15
54D_2F_Bd-37E_2F_Bd	-0.0107	-0.0279713	0.00657132	8.00E-01
54D_Bd-37E_2F_Bd	0.0972	0.07992868	0.11447132	9.21E-15
54D_Jliv10_Bd-37E_2F_Bd	0.0115	-0.0057713	0.02877132	6.85E-01
54D_JlivBTP_Bd-37E_2F_Bd	0.0238	0.00652868	0.04107132	2.50E-04
Jliv10_Bd-37E_2F_Bd	0.07293333	0.05518874	0.09067793	9.21E-15
Jliv10_JlivBTP_Bd-37E_2F_Bd	0.013225	-0.005094	0.031544	5.30E-01
JlivBTP_Bd-37E_2F_Bd	0.032175	0.013856	0.050494	2.76E-07
Live_Bd-37E_2F_Bd	0.1033	0.08045219	0.12614781	9.21E-15
37E_Bd-37E_54D_Bd	0.0054	-0.0118713	0.02267132	1.00E+00
37E_Jliv10_Bd-37E_54D_Bd	-0.0039	-0.0211713	0.01337132	1.00E+00
37E_JlivBTP_Bd-37E_54D_Bd	0.007	-0.0102713	0.02427132	9.97E-01
54D_20D_Bd-37E_54D_Bd	0.124925	0.106606	0.143244	9.21E-15
54D_2F_Bd-37E_54D_Bd	-0.0081	-0.0253713	0.00917132	9.84E-01
54D_Bd-37E_54D_Bd	0.0998	0.08252868	0.11707132	9.21E-15
54D_Jliv10_Bd-37E_54D_Bd	0.0141	-0.0031713	0.03137132	2.88E-01
54D_JlivBTP_Bd-37E_54D_Bd	0.0264	0.00912868	0.04367132	1.92E-05
Jliv10_Bd-37E_54D_Bd	0.07553333	0.05778874	0.09327793	9.21E-15

Jliv10_JlivBTP_Bd-37E_54D_Bd	0.015825	-0.002494	0.034144	1.96E-01
JlivBTP_Bd-37E_54D_Bd	0.034775	0.016456	0.053094	1.63E-08
Live_Bd-37E_54D_Bd	0.1059	0.08305219	0.12874781	9.21E-15
37E_Jliv10_Bd-37E_Bd	-0.0093	-0.0265713	0.00797132	9.35E-01
37E_JlivBTP_Bd-37E_Bd	0.0016	-0.0156713	0.01887132	1.00E+00
54D_20D_Bd-37E_Bd	0.119525	0.101206	0.137844	9.21E-15
54D_2F_Bd-37E_Bd	-0.0135	-0.0307713	0.00377132	3.70E-01
54D_Bd-37E_Bd	0.0944	0.07712868	0.11167132	9.21E-15
54D_Jliv10_Bd-37E_Bd	0.0087	-0.0085713	0.02597132	9.66E-01
54D_JlivBTP_Bd-37E_Bd	0.021	0.00372868	0.03827132	3.04E-03
Jliv10_Bd-37E_Bd	0.07013333	0.05238874	0.08787793	9.21E-15
Jliv10_JlivBTP_Bd-37E_Bd	0.010425	-0.007894	0.028744	8.95E-01
JlivBTP_Bd-37E_Bd	0.029375	0.011056	0.047694	4.98E-06
Live_Bd-37E_Bd	0.1005	0.07765219	0.12334781	9.21E-15
37E_JlivBTP_Bd-37E_Jliv10_Bd	0.0109	-0.0063713	0.02817132	7.73E-01
54D_20D_Bd-37E_Jliv10_Bd	0.128825	0.110506	0.147144	9.21E-15
54D_2F_Bd-37E_Jliv10_Bd	-0.0042	-0.0214713	0.01307132	1.00E+00
54D_Bd-37E_Jliv10_Bd	0.1037	0.08642868	0.12097132	9.21E-15
54D_Jliv10_Bd-37E_Jliv10_Bd	0.018	0.00072868	0.03527132	3.06E-02
54D_JlivBTP_Bd-37E_Jliv10_Bd	0.0303	0.01302868	0.04757132	2.87E-07
Jliv10_Bd-37E_Jliv10_Bd	0.07943333	0.06168874	0.09717793	9.21E-15
Jliv10_JlivBTP_Bd-37E_Jliv10_Bd	0.019725	0.001406	0.038044	2.00E-02
JlivBTP_Bd-37E_Jliv10_Bd	0.038675	0.020356	0.056994	1.86E-10
Live_Bd-37E_Jliv10_Bd	0.1098	0.08695219	0.13264781	9.21E-15

54D_20D_Bd-37E_JlivBTP_Bd	0.117925	0.099606	0.136244	9.21E-15
54D_2F_Bd-37E_JlivBTP_Bd	-0.0151	-0.0323713	0.00217132	1.79E-01
54D_Bd-37E_JlivBTP_Bd	0.0928	0.07552868	0.11007132	9.21E-15
54D_Jliv10_Bd-37E_JlivBTP_Bd	0.0071	-0.0101713	0.02437132	9.97E-01
54D_JlivBTP_Bd-37E_JlivBTP_Bd	0.0194	0.00212868	0.03667132	1.10E-02
Jliv10_Bd-37E_JlivBTP_Bd	0.06853333	0.05078874	0.08627793	9.21E-15
Jliv10_JlivBTP_Bd-37E_JlivBTP_Bd	0.008825	-0.009494	0.027144	9.79E-01
JlivBTP_Bd-37E_JlivBTP_Bd	0.027775	0.009456	0.046094	2.39E-05
Live_Bd-37E_JlivBTP_Bd	0.0989	0.07605219	0.12174781	9.21E-15
54D_2F_Bd-54D_20D_Bd	-0.133025	-0.151344	-0.114706	9.21E-15
54D_Bd-54D_20D_Bd	-0.025125	-0.043444	-0.006806	2.77E-04
54D_Jliv10_Bd-54D_20D_Bd	-0.110825	-0.129144	-0.092506	9.21E-15
54D_JlivBTP_Bd-54D_20D_Bd	-0.098525	-0.116844	-0.080206	9.21E-15
Jliv10_Bd-54D_20D_Bd	-0.0493917	-0.0681575	-0.0306258	2.20E-13
Jliv10_JlivBTP_Bd-54D_20D_Bd	-0.1091	-0.1284099	-0.0897901	9.21E-15
JlivBTP_Bd-54D_20D_Bd	-0.09015	-0.1094599	-0.0708401	9.21E-15
Live_Bd-54D_20D_Bd	-0.019025	-0.0426747	0.00462473	3.15E-01
54D_Bd-54D_2F_Bd	0.1079	0.09062868	0.12517132	9.21E-15
54D_Jliv10_Bd-54D_2F_Bd	0.0222	0.00492868	0.03947132	1.08E-03
54D_JlivBTP_Bd-54D_2F_Bd	0.0345	0.01722868	0.05177132	2.09E-09
Jliv10_Bd-54D_2F_Bd	0.08363333	0.06588874	0.10137793	9.21E-15
Jliv10_JlivBTP_Bd-54D_2F_Bd	0.023925	0.005606	0.042244	7.85E-04
JlivBTP_Bd-54D_2F_Bd	0.042875	0.024556	0.061194	1.40E-12

Live_Bd-54D_2F_Bd	0.114	0.09115219	0.13684781	9.21E-15
54D_Jliv10_Bd-54D_Bd	-0.0857	-0.1029713	-0.0684287	9.21E-15
54D_JlivBTP_Bd-54D_Bd	-0.0734	-0.0906713	-0.0561287	9.21E-15
Jliv10_Bd-54D_Bd	-0.0242667	-0.0420113	-0.0065221	2.96E-04
Jliv10_JlivBTP_Bd-54D_Bd	-0.083975	-0.102294	-0.065656	9.21E-15
JlivBTP_Bd-54D_Bd	-0.065025	-0.083344	-0.046706	9.88E-15
Live_Bd-54D_Bd	0.0061	-0.0167478	0.02894781	1.00E+00
54D_JlivBTP_Bd-54D_Jliv10_Bd	0.0123	-0.0049713	0.02957132	5.57E-01
Jliv10_Bd-54D_Jliv10_Bd	0.06143333	0.04368874	0.07917793	1.14E-14
Jliv10_JlivBTP_Bd-54D_Jliv10_Bd	0.001725	-0.016594	0.020044	1.00E+00
JlivBTP_Bd-54D_Jliv10_Bd	0.020675	0.002356	0.038994	1.02E-02
Live_Bd-54D_Jliv10_Bd	0.0918	0.06895219	0.11464781	9.21E-15
Jliv10_Bd-54D_JlivBTP_Bd	0.04913333	0.03138874	0.06687793	2.09E-13
Jliv10_JlivBTP_Bd-54D_JlivBTP_Bd	-0.010575	-0.028894	0.007744	8.82E-01
JlivBTP_Bd-54D_JlivBTP_Bd	0.008375	-0.009944	0.026694	9.88E-01
Live_Bd-54D_JlivBTP_Bd	0.0795	0.05665219	0.10234781	1.11E-14
Jliv10_JlivBTP_Bd-Jliv10_Bd	-0.0597083	-0.0784742	-0.0409425	5.46E-14
JlivBTP_Bd-Jliv10_Bd	-0.0407583	-0.0595242	-0.0219925	4.99E-11
Live_Bd-Jliv10_Bd	0.03036667	0.00715903	0.05357431	7.56E-04
JlivBTP_Bd-Jliv10_JlivBTP_Bd	0.01895	-0.0003599	0.03825992	6.15E-02
Live_Bd-Jliv10_JlivBTP_Bd	0.090075	0.06642527	0.11372473	9.21E-15
Live_Bd-JlivBTP_Bd	0.071125	0.04747527	0.09477473	1.38E-13

Supplementary Table 4: ANOVA with Tukey HSD for each grouping of bacteria, associated with results represented in Figure 3.

Treatment Comparison	Difference in mean value	Lower 95% confidence interval	Upper 95% confidence interval	Adjusted p-value (Tukey HSD)
20D_2F-20D	0.42817591	-0.2310249	1.08737674	0.62834404
20D_Jliv10-20D	0.16319633	-0.4960045	0.82239716	0.99998885
20D_JlivBTP-20D	0.08176348	-0.6552455	0.81877242	1
2F-20D	-0.0083597	-0.6675606	0.65084109	1
2F_Jliv10-20D	-0.0495361	-0.7087369	0.60966477	1
2F_JlivBTP-20D	0.05690945	-0.6022914	0.71611028	1
37E-20D	-0.0748807	-0.7340815	0.58432018	1
37E_20D-20D	0.08807723	-0.5711236	0.74727806	1
37E_2F-20D	0.09389766	-0.5653032	0.7530985	1
37E_54D-20D	0.41205297	-0.2471479	1.0712538	0.69090033
37E_Jliv10-20D	0.31452415	-0.3446767	0.97372498	0.95279709
37E_JlivBTP-20D	-0.0149788	-0.6741796	0.64422202	1
54D-20D	-0.1914946	-0.8506954	0.46770626	0.99987264
54D_20D-20D	0.13165793	-0.5275429	0.79085877	0.99999968
54D_2F-20D	0.23654604	-0.4226548	0.89574687	0.99776844
54D_Jliv10-20D	-0.0196769	-0.6788777	0.63952394	1
54D_JlivBTP-20D	-0.0127674	-0.6719682	0.64643347	1
Jliv10-20D	-0.045869	-0.7050698	0.61333187	1
Jliv10_JlivBTP-20D	0.67212034	0.01291951	1.33132117	0.04133835
JlivBTP-20D	0.52813115	-0.1310697	1.18733199	0.26725945
20D_Jliv10-20D_2F	-0.2649796	-0.9241804	0.39422125	0.99149416
20D_JlivBTP-20D_2F	-0.3464124	-1.0834214	0.39059651	0.95873119
2F-20D_2F	-0.4365357	-1.0957365	0.22266518	0.59523323
2F_Jliv10-20D_2F	-0.477712	-1.1369128	0.18148887	0.43459848
2F_JlivBTP-20D_2F	-0.3712665	-1.0304673	0.28793437	0.83093054
37E-20D_2F	-0.5030566	-1.1622574	0.15614428	0.34485573
37E_20D-20D_2F	-0.3400987	-0.9992995	0.31910216	0.90961888
37E_2F-20D_2F	-0.3342782	-0.9934791	0.32492259	0.92111854

37E_54D-20D_2F	-0.0161229	-0.6753238	0.6430779	1
37E_Jliv10-20D_2F	-0.1136518	-0.7728526	0.54554907	0.99999998
37E_JlivBTP-20D_2F	-0.4431547	-1.1023556	0.21604611	0.5689109
54D-20D_2F	-0.6196705	-1.2788713	0.03953036	0.08733246
54D_20D-20D_2F	-0.296518	-0.9557188	0.36268286	0.97264498
54D_2F-20D_2F	-0.1916299	-0.8508307	0.46757097	0.99987133
54D_Jliv10-20D_2F	-0.4478528	-1.1070536	0.21134803	0.55024367
54D_JlivBTP-20D_2F	-0.4409433	-1.1001441	0.21825756	0.57770696
Jliv10-20D_2F	-0.4740449	-1.1332457	0.18515597	0.44835206
Jliv10_JlivBTP-20D_2F	0.24394443	-0.4152564	0.90314527	0.99674332
JlivBTP-20D_2F	0.09995525	-0.5592456	0.75915608	1
20D_JlivBTP-20D_Jliv10	-0.0814328	-0.8184418	0.65557609	1
2F-20D_Jliv10	-0.1715561	-0.8307569	0.48764476	0.99997562
2F_Jliv10-20D_Jliv10	-0.2127324	-0.8719332	0.44646845	0.99943969
2F_JlivBTP-20D_Jliv10	-0.1062869	-0.7654877	0.55291395	0.99999999
37E-20D_Jliv10	-0.238077	-0.8972778	0.42112386	0.9975824
37E_20D-20D_Jliv10	-0.0751191	-0.7343199	0.58408174	1
37E_2F-20D_Jliv10	-0.0692987	-0.7284995	0.58990217	1
37E_54D-20D_Jliv10	0.24885665	-0.4103442	0.90805748	0.99586463
37E_Jliv10-20D_Jliv10	0.15132782	-0.507873	0.81052865	0.9999967
37E_JlivBTP-20D_Jliv10	-0.1781751	-0.837376	0.48102569	0.99995644
54D-20D_Jliv10	-0.3546909	-1.0138917	0.30450994	0.87632268
54D_20D-20D_Jliv10	-0.0315384	-0.6907392	0.62766244	1
54D_2F-20D_Jliv10	0.07334971	-0.5858511	0.73255055	1
54D_Jliv10-20D_Jliv10	-0.1828732	-0.8420741	0.47632761	0.99993551

54D_JlivBTP-20D_Jliv10	-0.1759637	-0.8351645	0.48323714	0.99996399
Jliv10-20D_Jliv10	-0.2090653	-0.8682661	0.45013555	0.99955819
Jliv10_JlivBTP-20D_Jliv10	0.50892401	-0.1502768	1.16812485	0.32561939
JlivBTP-20D_Jliv10	0.36493483	-0.294266	1.02413566	0.84918752
2F-20D_JlivBTP	-0.0901232	-0.8271322	0.64688571	1
2F_Jliv10-20D_JlivBTP	-0.1312995	-0.8683085	0.60570939	0.99999996
2F_JlivBTP-20D_JlivBTP	-0.024854	-0.761863	0.7121549	1
37E-20D_JlivBTP	-0.1566441	-0.8936531	0.5803648	0.99999908
37E_20D-20D_JlivBTP	0.00631375	-0.7306952	0.74332269	1
37E_2F-20D_JlivBTP	0.01213418	-0.7248748	0.74914312	1
37E_54D-20D_JlivBTP	0.33028949	-0.4067194	1.06729843	0.97360969
37E_Jliv10-20D_JlivBTP	0.23276066	-0.5042483	0.9697696	0.99958307
37E_JlivBTP-20D_JlivBTP	-0.0967423	-0.8337512	0.64026664	1
54D-20D_JlivBTP	-0.2732581	-1.010267	0.46375088	0.99666749
54D_20D-20D_JlivBTP	0.04989445	-0.6871145	0.78690339	1
54D_2F-20D_JlivBTP	0.15478256	-0.5822264	0.89179149	0.99999925
54D_Jliv10-20D_JlivBTP	-0.1014404	-0.8384493	0.63556856	1
54D_JlivBTP-20D_JlivBTP	-0.0945308	-0.8315398	0.64247809	1
Jliv10-20D_JlivBTP	-0.1276324	-0.8646414	0.6093765	0.99999997
Jliv10_JlivBTP-20D_JlivBTP	0.59035686	-0.1466521	1.32736579	0.26754415
JlivBTP-20D_JlivBTP	0.44636767	-0.2906413	1.18337661	0.7383818
2F_Jliv10-2F	-0.0411763	-0.7003771	0.61802452	1
2F_JlivBTP-2F	0.06526919	-0.5939316	0.72447002	1
37E-2F	-0.0665209	-0.7257217	0.59267993	1
37E_20D-2F	0.09643698	-0.5627639	0.75563781	1

37E_2F-2F	0.10225741	-0.5569434	0.76145824	1
37E_54D-2F	0.42041272	-0.2387881	1.07961355	0.65875488
37E_Jliv10-2F	0.32288389	-0.3363169	0.98208472	0.94074129
37E_JlivBTP-2F	-0.0066191	-0.6658199	0.65258177	1
54D-2F	-0.1831348	-0.8423357	0.47606601	0.99993412
54D_20D-2F	0.14001768	-0.5191832	0.79921851	0.99999909
54D_2F-2F	0.24490579	-0.414295	0.90410662	0.99658492
54D_Jliv10-2F	-0.0113171	-0.670518	0.64788368	1
54D_JlivBTP-2F	-0.0044076	-0.6636084	0.65479322	1
Jliv10-2F	-0.0375092	-0.69671	0.62169162	1
Jliv10_JlivBTP-2F	0.68048009	0.02127926	1.33968092	0.03648078
JlivBTP-2F	0.5364909	-0.1227099	1.19569173	0.24415855
2F_JlivBTP-2F_Jliv10	0.10644551	-0.5527553	0.76564634	0.99999999
37E-2F_Jliv10	-0.0253446	-0.6845454	0.63385624	1
37E_20D-2F_Jliv10	0.13761329	-0.5215875	0.79681412	0.99999932
37E_2F-2F_Jliv10	0.14343372	-0.5157671	0.80263456	0.99999864
37E_54D-2F_Jliv10	0.46158903	-0.1976118	1.12078986	0.4961568
37E_Jliv10-2F_Jliv10	0.3640602	-0.2951406	1.02326104	0.85162197
37E_JlivBTP-2F_Jliv10	0.03455725	-0.6246436	0.69375808	1
54D-2F_Jliv10	-0.1419585	-0.8011593	0.51724232	0.99999885
54D_20D-2F_Jliv10	0.18119399	-0.4780068	0.84039482	0.99994385
54D_2F-2F_Jliv10	0.2860821	-0.3731187	0.94528293	0.98080715
54D_Jliv10-2F_Jliv10	0.02985916	-0.6293417	0.68906	1
54D_JlivBTP-2F_Jliv10	0.0367687	-0.6224321	0.69596953	1
Jliv10-2F_Jliv10	0.0036671	-0.6555337	0.66286793	1
Jliv10_JlivBTP-2F_Jliv10	0.7216564	0.06245557	1.38085723	0.0193184
JlivBTP-2F_Jliv10	0.57766721	-0.0815336	1.23686805	0.15085046
37E-2F_JlivBTP	-0.1317901	-0.7909909	0.52741074	0.99999967
37E_20D-2F_JlivBTP	0.03116779	-0.628033	0.69036862	1

37E_2F-2F_JlivBTP	0.03698822	-0.6222126	0.69618905	1
37E_54D-2F_JlivBTP	0.35514353	-0.3040573	1.01434436	0.87518824
37E_Jliv10-2F_JlivBTP	0.2576147	-0.4015861	0.91681553	0.99381062
37E_JlivBTP-2F_JlivBTP	-0.0718883	-0.7310891	0.58731257	1
54D-2F_JlivBTP	-0.248404	-0.9076048	0.41079682	0.99595307
54D_20D-2F_JlivBTP	0.07474849	-0.5844523	0.73394932	1
54D_2F-2F_JlivBTP	0.17963659	-0.4795642	0.83883743	0.9999507
54D_Jliv10-2F_JlivBTP	-0.0765863	-0.7357872	0.58261449	1
54D_JlivBTP-2F_JlivBTP	-0.0696768	-0.7288776	0.58952402	1
Jliv10-2F_JlivBTP	-0.1027784	-0.7619792	0.55642243	1
Jliv10_JlivBTP-2F_JlivBTP	0.61521089	-0.0439899	1.27441173	0.09277152
JlivBTP-2F_JlivBTP	0.47122171	-0.1879791	1.13042254	0.45904854
37E_20D-37E	0.16295788	-0.4962429	0.82215872	0.99998911
37E_2F-37E	0.16877831	-0.4904225	0.82797915	0.99998109
37E_54D-37E	0.48693362	-0.1722672	1.14613445	0.4007976
37E_Jliv10-37E	0.3894048	-0.269796	1.04860563	0.77291833
37E_JlivBTP-37E	0.05990184	-0.599299	0.71910267	1
54D-37E	-0.1166139	-0.7758148	0.54258691	0.99999996
54D_20D-37E	0.20653858	-0.4526622	0.86573942	0.99962649
54D_2F-37E	0.31142669	-0.3477741	0.97062752	0.95678554
54D_Jliv10-37E	0.05520375	-0.6039971	0.71440459	1
54D_JlivBTP-37E	0.06211329	-0.5970875	0.72131412	1
Jliv10-37E	0.02901169	-0.6301891	0.68821252	1
Jliv10_JlivBTP-37E	0.74700099	0.08780016	1.40620182	0.01287101
JlivBTP-37E	0.60301181	-0.056189	1.26221264	0.10913797
37E_2F-37E_20D	0.00582043	-0.6533804	0.66502126	1
37E_54D-37E_20D	0.32397574	-0.3352251	0.98317657	0.93902327

37E_Jliv10-37E_20D	0.22644691	-0.4327539	0.88564774	0.99871727
37E_JlivBTP-37E_20D	-0.103056	-0.7622569	0.55614479	1
54D-37E_20D	-0.2795718	-0.9387726	0.37962903	0.98485046
54D_20D-37E_20D	0.0435807	-0.6156201	0.70278153	1
54D_2F-37E_20D	0.14846881	-0.510732	0.80766964	0.99999759
54D_Jliv10-37E_20D	-0.1077541	-0.766955	0.5514467	0.99999999
54D_JlivBTP-37E_20D	-0.1008446	-0.7600454	0.55835624	1
Jliv10-37E_20D	-0.1339462	-0.793147	0.52525464	0.99999957
Jliv10_JlivBTP-37E_20D	0.58404311	-0.0751577	1.24324394	0.13930988
JlivBTP-37E_20D	0.44005392	-0.2191469	1.09925475	0.58124454
37E_54D-37E_2F	0.31815531	-0.3410455	0.97735614	0.94779601
37E_Jliv10-37E_2F	0.22062648	-0.4385744	0.87982731	0.99908723
37E_JlivBTP-37E_2F	-0.1088765	-0.7680773	0.55032435	0.99999999
54D-37E_2F	-0.2853922	-0.9445931	0.3738086	0.98127142
54D_20D-37E_2F	0.03776027	-0.6214406	0.6969611	1
54D_2F-37E_2F	0.14264837	-0.5165525	0.80184921	0.99999876
54D_Jliv10-37E_2F	-0.1135746	-0.7727754	0.54562627	0.99999998
54D_JlivBTP-37E_2F	-0.106665	-0.7658659	0.5525358	0.99999999
Jliv10-37E_2F	-0.1397666	-0.7989675	0.51943421	0.99999912
Jliv10_JlivBTP-37E_2F	0.57822268	-0.0809782	1.23742351	0.14981574
JlivBTP-37E_2F	0.43423349	-0.2249673	1.09343432	0.60437519
37E_Jliv10-37E_54D	-0.0975288	-0.7567297	0.56167201	1
37E_JlivBTP-37E_54D	-0.4270318	-1.0862326	0.23216905	0.63285152
54D-37E_54D	-0.6035475	-1.2627484	0.05565329	0.10837155
54D_20D-37E_54D	-0.280395	-0.9395959	0.37880579	0.98437972

54D_2F-37E_54D	-0.1755069	-0.8347078	0.4836939	0.99996539
54D_Jliv10-37E_54D	-0.4317299	-1.0909307	0.22747096	0.61429977
54D_JlivBTP-37E_54D	-0.4248203	-1.0840212	0.2343805	0.64154124
Jliv10-37E_54D	-0.4579219	-1.1171228	0.2012789	0.51049013
Jliv10_JlivBTP-37E_54D	0.26006737	-0.3991335	0.9192682	0.99310502
JlivBTP-37E_54D	0.11607818	-0.5431226	0.77527901	0.99999996
37E_JlivBTP-37E_Jliv10	-0.329503	-0.9887038	0.32969787	0.92980222
54D-37E_Jliv10	-0.5060187	-1.1652195	0.15318212	0.33506532
54D_20D-37E_Jliv10	-0.1828662	-0.842067	0.47633462	0.99993555
54D_2F-37E_Jliv10	-0.0779781	-0.7371789	0.58122273	1
54D_Jliv10-37E_Jliv10	-0.334201	-0.9934019	0.32499979	0.92126428
54D_JlivBTP-37E_Jliv10	-0.3272915	-0.9864923	0.33190932	0.93359738
Jliv10-37E_Jliv10	-0.3603931	-1.0195939	0.29880773	0.86159231
Jliv10_JlivBTP-37E_Jliv10	0.3575962	-0.3016046	1.01679703	0.86893625
JlivBTP-37E_Jliv10	0.21360701	-0.4455938	0.87280784	0.99940764
54D-37E_JlivBTP	-0.1765158	-0.8357166	0.48268507	0.99996222
54D_20D-37E_JlivBTP	0.14663675	-0.5125641	0.80583758	0.99999803
54D_2F-37E_JlivBTP	0.25152485	-0.407676	0.91072568	0.99530997
54D_Jliv10-37E_JlivBTP	-0.0046981	-0.6638989	0.65450275	1
54D_JlivBTP-37E_JlivBTP	0.00221145	-0.6569894	0.66141228	1
Jliv10-37E_JlivBTP	-0.0308901	-0.690091	0.62831069	1
Jliv10_JlivBTP-37E_JlivBTP	0.68709915	0.02789832	1.34629999	0.03300921
JlivBTP-37E_JlivBTP	0.54310997	-0.1160909	1.2023108	0.22687598
54D_20D-54D	0.3231525	-0.3360483	0.98235334	0.94032176

54D_2F-54D	0.42804061	-0.2311602	1.08724144	0.62887745
54D_Jliv10-54D	0.17181767	-0.4873832	0.83101851	0.99997504
54D_JlivBTP-54D	0.17872721	-0.4804736	0.83792804	0.99995435
Jliv10-54D	0.14562561	-0.5135752	0.80482644	0.99999825
Jliv10_JlivBTP-54D	0.86361491	0.20441408	1.52281574	0.00178076
JlivBTP-54D	0.71962572	0.06042489	1.37882656	0.01994814
54D_2F-54D_20D	0.10488811	-0.5543127	0.76408894	0.99999999
54D_Jliv10-54D_20D	-0.1513348	-0.8105357	0.507866	0.99999669
54D_JlivBTP-54D_20D	-0.1444253	-0.8036261	0.51477554	0.99999847
Jliv10-54D_20D	-0.1775269	-0.8367277	0.48167394	0.99995879
Jliv10_JlivBTP-54D_20D	0.54046241	-0.1187384	1.19966324	0.23368181
JlivBTP-54D_20D	0.39647322	-0.2627276	1.05567405	0.74829575
54D_Jliv10-54D_2F	-0.2562229	-0.9154238	0.4029779	0.99418393
54D_JlivBTP-54D_2F	-0.2493134	-0.9085142	0.40988743	0.99577375
Jliv10-54D_2F	-0.282415	-0.9416158	0.37678583	0.98317605
Jliv10_JlivBTP-54D_2F	0.4355743	-0.2236265	1.09477513	0.5990523
JlivBTP-54D_2F	0.29158512	-0.3676157	0.95078595	0.97677807
54D_JlivBTP-54D_Jliv10	0.00690953	-0.6522913	0.66611036	1
Jliv10-54D_Jliv10	-0.0261921	-0.6853929	0.63300877	1
Jliv10_JlivBTP-54D_Jliv10	0.69179724	0.0325964	1.35099807	0.03073113
JlivBTP-54D_Jliv10	0.54780805	-0.1113928	1.20700888	0.21514961
Jliv10-54D_JlivBTP	-0.0331016	-0.6923024	0.62609924	1
Jliv10_JlivBTP-54D_JlivBTP	0.6848877	0.02568687	1.34408854	0.03413404
JlivBTP-54D_JlivBTP	0.54089852	-0.1183023	1.20009935	0.23255093
Jliv10_JlivBTP-Jliv10	0.7179893	0.05878847	1.37719013	0.02046947
JlivBTP-Jliv10	0.57400011	-0.0852007	1.23320094	0.15782435

JlivBTP-Jliv10_JlivBTP	-0.1439892	-0.80319	0.51521165	0.99999855

Supplementary Table 5: ANOVA with Tukey HSD for each grouping of bacteria, associated with results represented in Supplementary Figure 1.

Treatment	ANOVA p-value	ANOVA F-value	Tukey HSD comparison	Tukey HSD difference in mean value	Tukey HSD lower 95% confidence interval	Tukey HSD upper 95% confidence interval	Tukey HSD adjusted p-value
20D_2F	2.00E-16	204.8	20D_Bd-20D_2F_Bd	0.0083	-0.0022254	0.01882545	0.1624913
			2F_Bd-20D_2F_Bd	-0.0041	-0.0146254	0.00642545	0.7164278
			Live_Bd-20D_2F_Bd	0.1146	0.10067614	0.12852386	0
			2F_Bd-20D_Bd	-0.0124	-0.0229254	-0.0018746	0.0160526
			Live_Bd-20D_Bd	0.1063	0.09237614	0.12022386	0
			Live_Bd-2F_Bd	0.1187	0.10477614	0.13262386	0
20D_54D	2.00E-16	137	54D_20D_Bd-20D_Bd	0.125325	0.1065088	0.1441412	0
			54D_Bd-20D_Bd	0.1002	0.08245991	0.11794009	0
			Live_Bd-20D_Bd	0.1063	0.08283207	0.12976793	0
			54D_Bd-54D_20D_Bd	-0.025125	-0.0439412	-0.0063088	0.0056033
			Live_Bd-54D_20D_Bd	-0.019025	-0.0433166	0.00526661	0.1658524
			Live_Bd-54D_Bd	0.0061	-0.0173679	0.02956793	0.8924084
20D_Jliv10	3.36E-11	46.48	20D_Jliv10_Bd-20D_Bd	0.046	0.02443296	0.06756704	0.0000153
			Jliv10_Bd-20D_Bd	0.07593333	0.0537753	0.09809136	0
			Live_Bd-20D_Bd	0.1063	0.07776948	0.13483052	0
			Jliv10_Bd-20D_Jliv10_Bd	0.02993333	0.0077753	0.05209136	0.0049497
			Live_Bd-20D_Jliv10_Bd	0.0603	0.03176948	0.08883052	0.0000177
			Live_Bd-Jliv10_Bd	0.03036667	0.00138682	0.05934651	0.0373013
20D_JlivBTP	4.92E-14	82.83	20D_JlivBTP_Bd-20D_Bd	0.0066	-0.0082752	0.02147523	0.6249395
			JlivBTP_Bd-20D_Bd	0.035175	0.01939743	0.05095257	0.0000083
			Live_Bd-20D_Bd	0.1063	0.08662192	0.12597808	0
			JlivBTP_Bd-20D_JlivBTP_Bd	0.028575	0.01279743	0.04435257	0.0001802
			Live_Bd-20D_JlivBTP_Bd	0.0997	0.08002192	0.11937808	0
			Live_Bd-JlivBTP_Bd	0.071125	0.05075625	0.09149375	0
20D_37E	2.00E-16	123.4	37E_20D_Bd-20D_Bd	0.0001	-0.0123241	0.01252408	0.9999961
			37E_Bd-20D_Bd	0.0058	-0.0066241	0.01822408	0.5889182
			Live_Bd-20D_Bd	0.1063	0.08986449	0.12273551	0
			37E_Bd-37E_20D_Bd	0.0057	-0.0067241	0.01812408	0.6023956
			Live_Bd-37E_20D_Bd	0.1062	0.08976449	0.12263551	0
			Live_Bd-37E_Bd	0.1005	0.08406449	0.11693551	0
2F_54D	2.00E-16	278	54D_2F_Bd-2F_Bd	0.0047	-0.0088463	0.01824633	0.7818648
			54D_Bd-2F_Bd	0.1126	0.09905367	0.12614633	0

			Live_Bd-2F_Bd	0.1187	0.10077989	0.13662011	0
			54D_Bd-54D_2F_Bd	0.1079	0.09435367	0.12144633	0
			Live_Bd-54D_2F_Bd	0.114	0.09607989	0.13192011	0
			Live_Bd-54D_Bd	0.0061	-0.0118201	0.02402011	0.7914321
2F_Jliv10	2.00E-16	176	2F_Jliv10_Bd-2F_Bd	-0.0167333	-0.0331078	-0.0003589	0.0437935
			Jliv10_Bd-2F_Bd	0.08833333	0.07195891	0.10470776	0
			Live_Bd-2F_Bd	0.1187	0.09761641	0.13978359	0
			Jliv10_Bd-2F_Jliv10_Bd	0.10506667	0.08826686	0.12186648	0
			Live_Bd-2F_Jliv10_Bd	0.13543333	0.1140177	0.15684897	0
			Live_Bd-Jliv10_Bd	0.03036667	0.00895103	0.0517823	0.0031385
2F_JlivBTP	2.00E-16	184.6	2F_JlivBTP_Bd-2F_Bd	-0.0004	-0.0122095	0.01140947	0.9997094
			JlivBTP_Bd-2F_Bd	0.047575	0.03504916	0.06010084	0
			Live_Bd-2F_Bd	0.1187	0.10307754	0.13432246	0
			JlivBTP_Bd-2F_JlivBTP_Bd	0.047975	0.03544916	0.06050084	0
			Live_Bd-2F_JlivBTP_Bd	0.1191	0.10347754	0.13472246	0
			Live_Bd-JlivBTP_Bd	0.071125	0.05495422	0.08729578	0
2F_37E	2.00E-16	138.2	37E_2F_Bd-2F_Bd	0.0154	0.00305692	0.02774308	0.0100259
			37E_Bd-2F_Bd	0.0182	0.00585692	0.03054308	0.0020031
			Live_Bd-2F_Bd	0.1187	0.10237164	0.13502836	0
			37E_Bd-37E_2F_Bd	0.0028	-0.0095431	0.01514308	0.9259084
			Live_Bd-37E_2F_Bd	0.1033	0.08697164	0.11962836	0
			Live_Bd-37E_Bd	0.1005	0.08417164	0.11682836	0
54D_Jliv10	1.96E-12	58.73	54D_Jliv10_Bd-54D_Bd	-0.0857	-0.1052638	-0.0661362	0
			Jliv10_Bd-54D_Bd	-0.0242667	-0.0443666	-0.0041668	0.0132894
			Live_Bd-54D_Bd	0.0061	-0.0197805	0.03198049	0.9174112
			Jliv10_Bd-54D_Jliv10_Bd	0.06143333	0.04133343	0.08153324	0
			Live_Bd-54D_Jliv10_Bd	0.0918	0.06591951	0.11768049	0
			Live_Bd-Jliv10_Bd	0.03036667	0.00407859	0.05665475	0.0187852
54D_JlivBTP	2.46E-14	87.52	54D_JlivBTP_Bd-54D_Bd	-0.0734	-0.0885814	-0.0582186	0
			JlivBTP_Bd-54D_Bd	-0.065025	-0.0811273	-0.0489227	0
			Live_Bd-54D_Bd	0.0061	-0.013983	0.02618305	0.8401052
			JlivBTP_Bd-54D_JlivBTP_Bd	0.008375	-0.0077273	0.02447726	0.4978124
			Live_Bd-54D_JlivBTP_Bd	0.0795	0.05941695	0.09958305	0
			Live_Bd-JlivBTP_Bd	0.071125	0.05033707	0.09191293	0
54D_37E	2.00E-16	165.5	37E_Bd-37E_54D_Bd	0.0054	-0.0101043	0.02090433	0.779902

			54D_Bd-37E_54D_Bd	0.0998	0.08429567	0.11530433	0
			Live_Bd-37E_54D_Bd	0.1059	0.0853897	0.1264103	0
			54D_Bd-37E_Bd	0.0944	0.07889567	0.10990433	0
			Live_Bd-37E_Bd	0.1005	0.0799897	0.1210103	0
			Live_Bd-54D_Bd	0.0061	-0.0144103	0.0266103	0.849839
Jliv10_JlivBTP	6.44E-10	42.05	Jliv10_JlivBTP_Bd-Jliv10_Bd	-0.0597083	-0.080129	-0.0392876	0.0000001
			JlivBTP_Bd-Jliv10_Bd	-0.0407583	-0.061179	-0.0203376	0.0000592
			Live_Bd-Jliv10_Bd	0.03036667	0.0051125	0.05562083	0.0141787
			JlivBTP_Bd-Jliv10_JlivBTP_Bd	0.01895	-0.0020627	0.03996274	0.0877778
			Live_Bd-Jliv10_JlivBTP_Bd	0.090075	0.06433976	0.11581024	0
			Live_Bd-JlivBTP_Bd	0.071125	0.04538976	0.09686024	0.0000003
Jliv10_37E	2.07E-15	100.3	37E_Jliv10_Bd-37E_Bd	-0.0093	-0.0263281	0.00772809	0.4574186
			Jliv10_Bd-37E_Bd	0.07013333	0.05263864	0.08762803	0
			Live_Bd-37E_Bd	0.1005	0.07797396	0.12302604	0
			Jliv10_Bd-37E_Jliv10_Bd	0.07943333	0.06193864	0.09692803	0
			Live_Bd-37E_Jliv10_Bd	0.1098	0.08727396	0.13232604	0
			Live_Bd-Jliv10_Bd	0.03036667	0.00748586	0.05324747	0.0058453
JlivBTP_37E	1.95E-14	89.14	37E_JlivBTP_Bd-37E_Bd	0.0016	-0.0121688	0.0153688	0.9887037
			JlivBTP_Bd-37E_Bd	0.029375	0.01477098	0.04397902	0.0000411
			Live_Bd-37E_Bd	0.1005	0.08228559	0.11871441	0
			JlivBTP_Bd-37E_JlivBTP_Bd	0.027775	0.01317098	0.04237902	0.0000922
			Live_Bd-37E_JlivBTP_Bd	0.0989	0.08068559	0.11711441	0
			Live_Bd-JlivBTP_Bd	0.071125	0.0522713	0.0899787	0

Supplementary Table 6: ANOVA with Tukey HSD for each grouping of bacteria, associated with results represented in Supplementary Figure 3.

Treatment	ANOVA p-value	ANOVA F-value	Tukey HSD comparison	Tukey HSD difference in mean value	Tukey HSD lower 95% confidence interval	Tukey HSD upper 95% confidence interval	Tukey HSD adjusted p-value
20D_2F	2.00E-16	127.5	20D_Bd-20D_2F_Bd	0.0174	-0.0061376	0.04093756	0.209251
			2F_Bd-20D_2F_Bd	0.0056	-0.0179376	0.02913756	0.917399
			Live_Bd-20D_2F_Bd	0.158525	0.13355965	0.18349035	0
			2F_Bd-20D_Bd	-0.0118	-0.0353376	0.01173756	0.5360449
			Live_Bd-20D_Bd	0.141125	0.11615965	0.16609035	0
			Live_Bd-2F_Bd	0.152925	0.12795965	0.17789035	0
20D_54D	2.00E-16	106.4	54D_20D_Bd-20D_Bd	0.17888889	0.15014176	0.20763602	0
			54D_Bd-20D_Bd	0.0882	0.0602196	0.1161804	0
			Live_Bd-20D_Bd	0.141125	0.1114473	0.1708027	0
			54D_Bd-54D_20D_Bd	-0.0906889	-0.119436	-0.0619418	0
			Live_Bd-54D_20D_Bd	-0.0377639	-0.0681655	-0.0073622	0.0101696
			Live_Bd-54D_Bd	0.052925	0.0232473	0.0826027	0.0001747
20D_Jliv10	1.34E-14	72.39	20D_Jliv10_Bd-20D_Bd	0.03545556	0.00710963	0.06380148	0.0095732
			Jliv10_Bd-20D_Bd	0.0013	-0.0262899	0.0288899	0.9992446
			Live_Bd-20D_Bd	0.141125	0.11186149	0.17038851	0
			Jliv10_Bd-20D_Jliv10_Bd	-0.0341556	-0.0625015	-0.0058096	0.0131567
			Live_Bd-20D_Jliv10_Bd	0.10566944	0.07569209	0.1356468	0
			Live_Bd-Jliv10_Bd	0.139825	0.11056149	0.16908851	0
20D_JlivBTP	7.23E-14	66.77	20D_JlivBTP_Bd-20D_Bd	0.0033	-0.0253936	0.03199362	0.9893128
			JlivBTP_Bd-20D_Bd	0.054925	0.02449082	0.08535918	0.0001544
			Live_Bd-20D_Bd	0.141125	0.11069082	0.17155918	0
			JlivBTP_Bd-20D_JlivBTP_Bd	0.051625	0.02119082	0.08205918	0.0003574
			Live_Bd-20D_JlivBTP_Bd	0.137825	0.10739082	0.16825918	0
			Live_Bd-JlivBTP_Bd	0.0862	0.05411956	0.11828044	0.0000002
20D_37E	2.00E-16	115.7	37E_20D_Bd-20D_Bd	-0.0051	-0.0284445	0.01824453	0.9344086
			37E_Bd-20D_Bd	-0.0012	-0.0245445	0.02214453	0.9990256
			Live_Bd-20D_Bd	0.141125	0.11636438	0.16588562	0
			37E_Bd-37E_20D_Bd	0.0039	-0.0194445	0.02724453	0.9689434
			Live_Bd-37E_20D_Bd	0.146225	0.12146438	0.17098562	0
			Live_Bd-37E_Bd	0.142325	0.11756438	0.16708562	0
2F_54D	2.00E-16	146.7	54D_2F_Bd-2F_Bd	-0.0072	-0.0306475	0.01624751	0.840167
			54D_Bd-2F_Bd	0.1	0.07655249	0.12344751	0
			Live_Bd-2F_Bd	0.152925	0.12805516	0.17779484	0
			54D_Bd-54D_2F_Bd	0.1072	0.08375249	0.13064751	0
			Live_Bd-54D_2F_Bd	0.160125	0.13525516	0.18499484	0

			Live_Bd-54D_Bd	0.052925	0.02805516	0.07779484	0.0000106
2F_Jliv10	2.00E-16	119.7	2F_Jliv10_Bd-2F_Bd	-0.0096	-0.034128	0.014928	0.7174334
			Jliv10_Bd-2F_Bd	0.0131	-0.011428	0.037628	0.4825183
			Live_Bd-2F_Bd	0.152925	0.12690913	0.1789409	0
			Jliv10_Bd-2F_Jliv10_Bd	0.0227	-0.001828	0.047228	0.0780137
			Live_Bd-2F_Jliv10_Bd	0.162525	0.13650913	0.1885409	0
			Live_Bd-Jliv10_Bd	0.139825	0.11380913	0.1658409	0
2F_JlivBTP	4.18E-16	96.33	2F_JlivBTP_Bd-2F_Bd	-0.0124	-0.0400181	0.01521809	0.6211806
			JlivBTP_Bd-2F_Bd	0.066725	0.03743159	0.09601841	0.0000038
			Live_Bd-2F_Bd	0.152925	0.12363159	0.18221841	0
			JlivBTP_Bd-2F_JlivBTP_Bd	0.079125	0.04983159	0.10841841	0.0000001
			Live_Bd-2F_JlivBTP_Bd	0.165325	0.13603159	0.19461841	0
			Live_Bd-JlivBTP_Bd	0.0862	0.05532203	0.11707797	0.0000001
2F_37E	2.00E-16	123.9	37E_2F_Bd-2F_Bd	0.0004	-0.0231601	0.02396006	0.9999646
			37E_Bd-2F_Bd	0.0106	-0.0129601	0.03416006	0.6217196
			Live_Bd-2F_Bd	0.152925	0.12793579	0.17791421	0
			37E_Bd-37E_2F_Bd	0.0102	-0.0133601	0.03376006	0.6499246
			Live_Bd-37E_2F_Bd	0.152525	0.12753579	0.17751421	0
			Live_Bd-37E_Bd	0.142325	0.11733579	0.16731421	0
54D_Jliv10	3.25E-13	55.74	54D_Jliv10_Bd-54D_Bd	-0.0555	-0.0853859	-0.0256141	0.0000932
			Jliv10_Bd-54D_Bd	-0.0869	-0.1167859	-0.0570141	0
			Live_Bd-54D_Bd	0.052925	0.02122619	0.08462381	0.0004112
			Jliv10_Bd-54D_Jliv10_Bd	-0.0314	-0.0612859	-0.0015141	0.0364368
			Live_Bd-54D_Jliv10_Bd	0.108425	0.07672619	0.14012381	0
			Live_Bd-Jliv10_Bd	0.139825	0.10812619	0.17152381	0
54D_JlivBTP	4.85E-12	48.79	54D_JlivBTP_Bd-54D_Bd	-0.0785	-0.1071695	-0.0498305	0.0000001
			JlivBTP_Bd-54D_Bd	-0.033275	-0.0636836	-0.0028664	0.0276414
			Live_Bd-54D_Bd	0.052925	0.02251644	0.08333356	0.0002541
			JlivBTP_Bd-54D_JlivBTP_Bd	0.045225	0.01481644	0.07563356	0.0017471
			Live_Bd-54D_JlivBTP_Bd	0.131425	0.10101644	0.16183356	0
			Live_Bd-JlivBTP_Bd	0.0862	0.05414656	0.11825344	0.0000002
54D_37E	2.00E-16	119.6	37E_Bd-37E_54D_Bd	0.0026	-0.0209183	0.02611831	0.9905776
			54D_Bd-37E_54D_Bd	0.092	0.06848169	0.11551831	0
			Live_Bd-37E_54D_Bd	0.144925	0.11998006	0.16986994	0
			54D_Bd-37E_Bd	0.0894	0.06588169	0.11291831	0
			Live_Bd-37E_Bd	0.142325	0.11738006	0.16726994	0
			Live_Bd-54D_Bd	0.052925	0.02798006	0.07786994	0.0000112
Jliv10_JlivBTP	1.43E-14	75.08	Jliv10_JlivBTP_Bd-Jliv10_Bd	-0.0178	-0.0469624	0.01136244	0.3641833
			JlivBTP_Bd-Jliv10_Bd	0.053625	0.02269357	0.08455643	0.0002679

			Live_Bd-Jliv10_Bd	0.139825	0.10889357	0.17075643	0
			JlivBTP_Bd-Jliv10_JlivBTP_Bd	0.071425	0.04049357	0.10235643	0.000003
			Live_Bd-Jliv10_JlivBTP_Bd	0.157625	0.12669357	0.18855643	0
			Live_Bd-JlivBTP_Bd	0.0862	0.05359541	0.11880459	0.0000002
Jliv10_37E	2.00E-16	102.7	37E_Jliv10_Bd-37E_Bd	-0.0133	-0.0386679	0.01206791	0.4983742
			Jliv10_Bd-37E_Bd	0.0025	-0.0228679	0.02786791	0.9932758
			Live_Bd-37E_Bd	0.142325	0.11541827	0.16923173	0
			Jliv10_Bd-37E_Jliv10_Bd	0.0158	-0.0095679	0.04116791	0.3485028
			Live_Bd-37E_Jliv10_Bd	0.155625	0.12871827	0.18253173	0
			Live_Bd-Jliv10_Bd	0.139825	0.11291827	0.16673173	0
JlivBTP_37E	5.27E-15	80.61	37E_JlivBTP_Bd-37E_Bd	-0.0086	-0.0361868	0.01898676	0.8327627
			JlivBTP_Bd-37E_Bd	0.056125	0.02686482	0.08538518	0.0000638
			Live_Bd-37E_Bd	0.142325	0.11306482	0.17158518	0
			JlivBTP_Bd-37E_JlivBTP_Bd	0.064725	0.03546482	0.09398518	0.0000064
			Live_Bd-37E_JlivBTP_Bd	0.150925	0.12166482	0.18018518	0
			Live_Bd-JlivBTP_Bd	0.0862	0.05535706	0.11704294	0.0000001

Supplementary Table 7: The T-test results from all comparisons between combinations and individual CFS trials from the separate-then-combined CFS trial. These results were used to determine if a pair worked additively, synergistically, or antagonistically.

Treatment	Outcome	t statistic	p-value	95% confidence intervals (lower, upper)	mean of the differences
20D_2F	Antagonistic	42.423	1.12E-11	(0.1282983, 0.1427517)	0.135525
20D_54D	Antagonistic	29.329	1.98E-09	(0.2139661, 0.2504839)	0.232225
20D_Jliv10	Antagonistic	23.69	1.07E-08	(0.1612275, 0.1960003)	0.1786139
20D_JlivBTP	Antagonistic	9.9321	2.24E-05	(0.06624898, 0.10765102)	0.08695
20D_37E	Antagonistic	53.076	1.50E-12	(0.1313764, 0.1430736)	0.137225
2F_54D	Antagonistic	13.336	3.12E-07	(0.03796852, 0.05348148)	0.045725
2F_Jliv10	Antagonistic	28.72	3.66E-10	(0.1199677, 0.1404823)	0.130225
2F_JlivBTP	Antagonistic	9.1498	3.83E-05	(0.05591393, 0.09488607)	0.0754
2F_37E	Antagonistic	32.759	1.13E-10	(0.1328691, 0.1525809)	0.142725
54D_Jliv10	Antagonistic	9.0056	8.49E-06	(0.06314311, 0.10550689)	0.084325
54D_JlivBTP	Additive	0.9533	0.3722	(-0.01225073, 0.02880073)	0.008275
54D_37E	Antagonistic	18.907	1.49E-08	(0.0443039, 0.0563461)	0.050325
Jliv10_JlivBTP	Antagonistic	11.099	1.07E-05	(0.05636578, 0.08688422)	0.071625
Jliv10_37E	Antagonistic	20.607	6.97E-09	(0.1126355, 0.1404145)	0.126525
JlivBTP_37E	Antagonistic	8.7844	4.99E-05	(0.05751507, 0.09988493)	0.0787

Supplementary Figure 1: Assay results from bacterial isolates grown together and separately in a multi-paneled figure showing each multi-strain pair treatment and its respective single-strain treatments. The mean *Bd* growth at 100 hours, measured as optical density, in an experiment testing CFS effect on *Bd* growth from bacteria grown in single- and multi-strain cultures. Color denotes whether the CFS was derived from one bacterial strain or a two-strain combination, or included no CFS (in the case of the live *Bd* control). Mean and standard error from an ANOVA with Tukey HSD test are shown here with significance groupings (letters) above each error bar (detailed statistical results are in Supplementary Table 5).

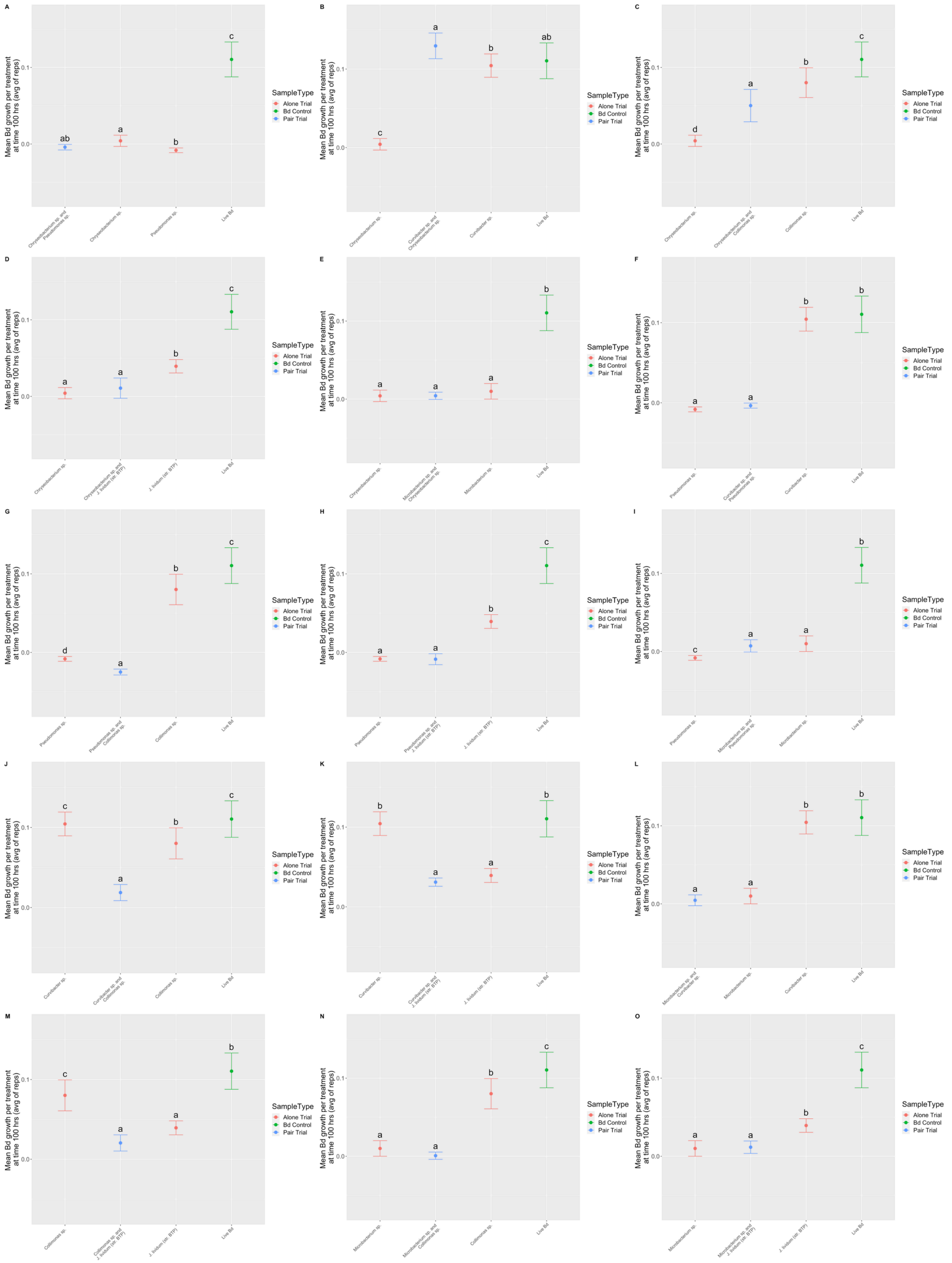
A
B
C
D
E
F
G
H
I
J
K
L
M
N
O
Mean Bd growth per treatment
at time 100 hrs (avg of reps)
0.0
0.1
SampleType
Alone Trial
Bd Control
Pair Trial
Chryseobacterium sp. and Pseudomonas sp.
Chryseobacterium sp.
Pseudomonas sp.
Live Bd
Curvibacter sp. and Chryseobacterium sp.
Curvibacter sp.
Chryseobacterium sp. and Collimonas sp.
Collimonas sp.
Chryseobacterium sp. and J. lividum (str. BTP)
J. lividum (str. BTP)
Microbacterium sp. and Chryseobacterium sp.
Microbacterium sp.
Curvibacter sp. and Pseudomonas sp.
Pseudomonas sp. and Collimonas sp.
Pseudomonas sp. and J. lividum (str. BTP)
Microbacterium sp. and Pseudomonas sp.
Curvibacter sp. and Collimonas sp.
Curvibacter sp. and J. lividum (str. BTP)
Microbacterium sp. and Curvibacter sp.
Collimonas sp. and J. lividum (str. BTP)
Microbacterium sp. and Collimonas sp.
Microbacterium sp. and J. lividum (str. BTP)

Supplementary Figure 2: The average bacterial carrying capacity (k), as estimated by growth curve modeling using optical density data. We tested six single microbes and 15 combinations (background color). Mean and standard error from an ANOVA with Tukey HSD test are shown here with significance groupings (letters) above each error bar (ANOVA p-value = 0.289).

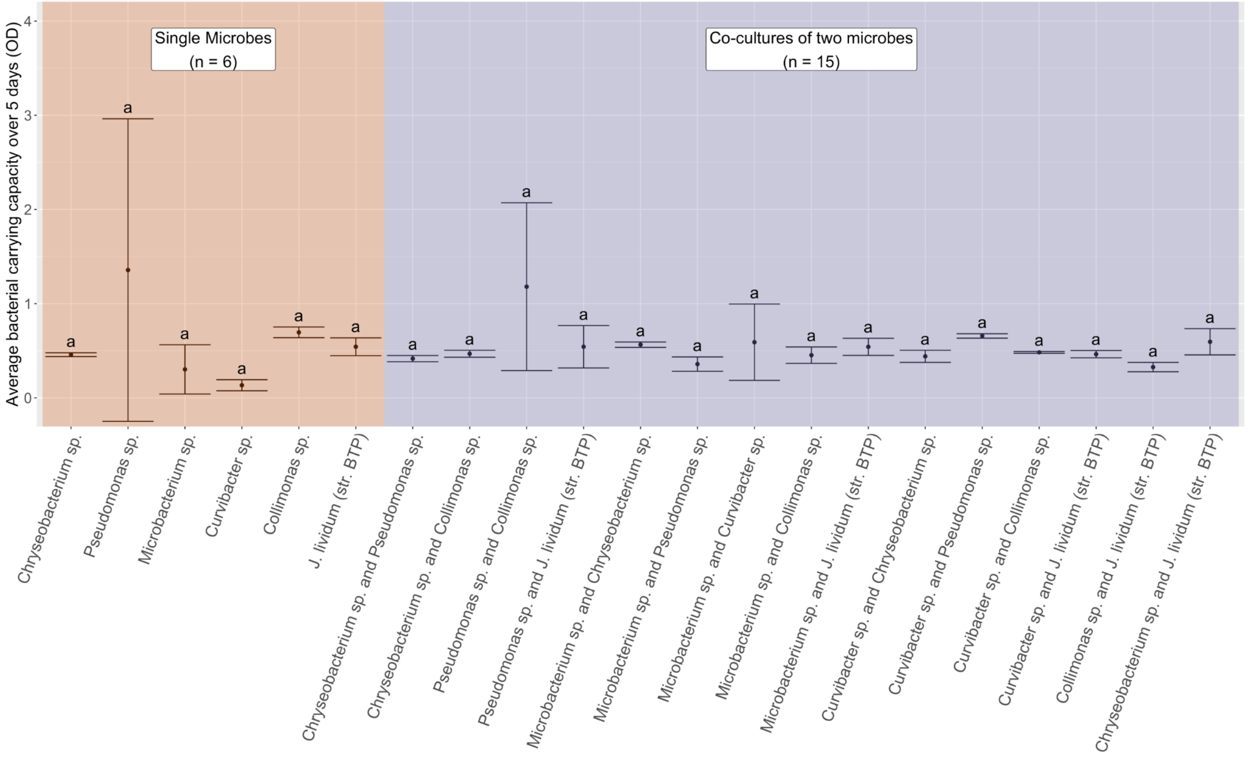

Supplementary Figure 3: Separate-then-combined assay results in a multi-paneled figure showing each multi-strain pair treatment and its respective single-strain treatments. The difference in *Bd* growth at 100 hours when grown with bacterial CFS compared to the live *Bd* control, measured as optical density, in an experiment testing separate-then-combined CFS effect on *Bd* growth (live *Bd* OD minus CFS treatment OD). This graph shows all the combinations tested in this assay type. Color denotes whether the CFS was derived from one bacterial strain or two-strain combinations or included no CFS (in the case of the live *Bd* control). Means and standard errors from an ANOVA with Tukey HSD test are shown here with significance groupings (letters) above each error bar (detailed statistical results are in Supplementary Table 6). Labels in the top right corner of each graph show whether a t-test pointed to an additive or antagonistic effect (there were no synergistic effects).

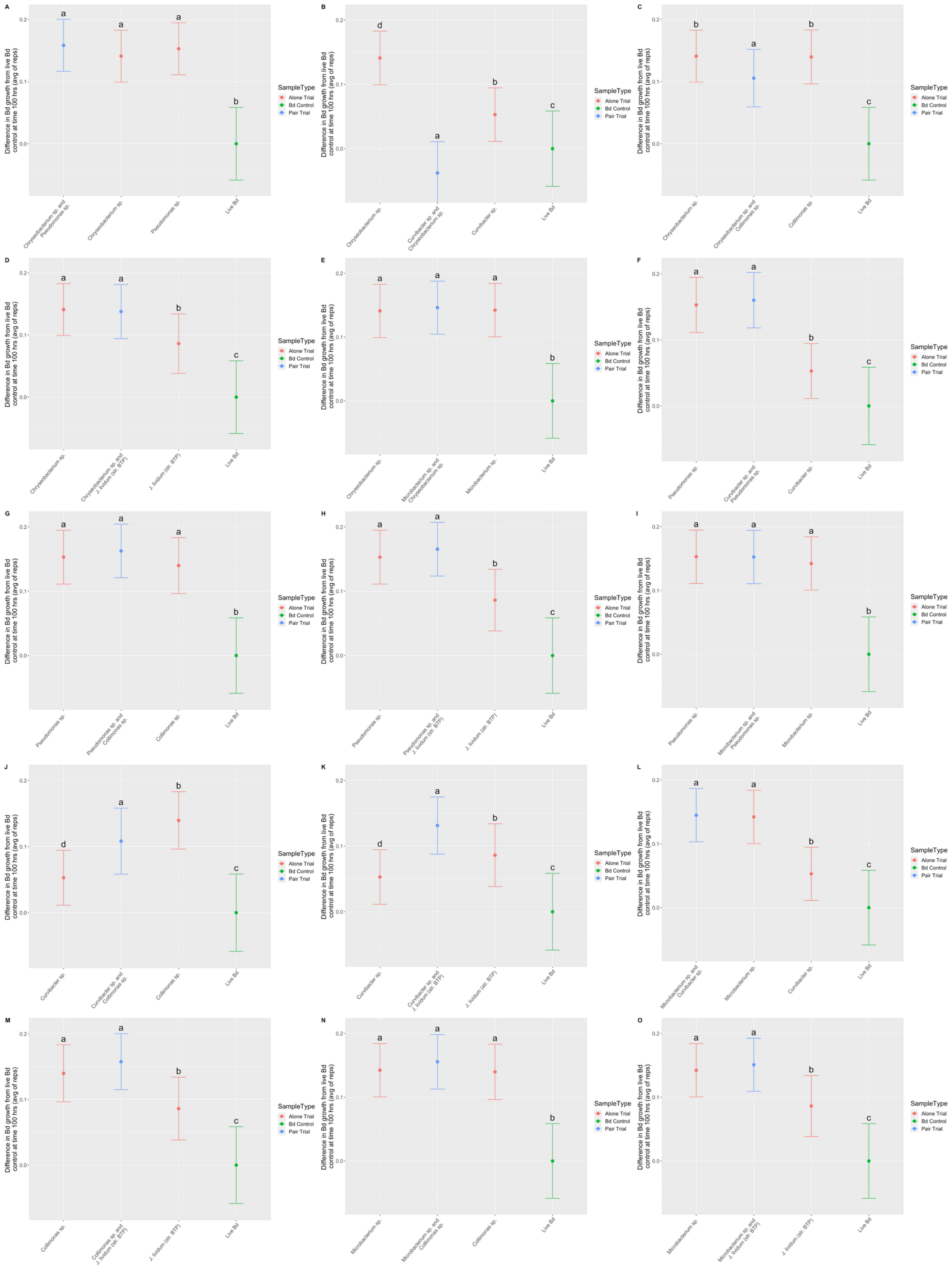
A
Difference in Bd growth from live Bd control at time 100 hrs (avg of reps)
SampleType
Alone Trial
Bd Control
Pair Trial
Chryseobacterium sp. and Pseudomonas sp.
Chryseobacterium sp.
Pseudomonas sp.
Live Bd
B
Chryseobacterium sp.
Curvibacter sp. and Chryseobacterium sp.
Curvibacter sp.
Live Bd
C
Chryseobacterium sp.
Chryseobacterium sp. and Collimonas sp.
Collimonas sp.
Live Bd
D
Chryseobacterium sp.
Chryseobacterium sp. and J. lividum (str. BTP)
J. lividum (str. BTP)
Live Bd
E
Chryseobacterium sp.
Microbacterium sp. and Chryseobacterium sp.
Microbacterium sp.
Live Bd
F
Pseudomonas sp.
Curvibacter sp. and Pseudomonas sp.
Curvibacter sp.
Live Bd
G
Pseudomonas sp.
Pseudomonas sp. and Collimonas sp.
Collimonas sp.
Live Bd
H
Pseudomonas sp.
Pseudomonas sp. and J. lividum (str. BTP)
J. lividum (str. BTP)
Live Bd
I
Pseudomonas sp.
Microbacterium sp. and Pseudomonas sp.
Microbacterium sp.
Live Bd
J
Curvibacter sp.
Curvibacter sp. and Collimonas sp.
Collimonas sp.
Live Bd
K
Curvibacter sp.
Curvibacter sp. and J. lividum (str. BTP)
J. lividum (str. BTP)
Live Bd
L
Microbacterium sp. and Curvibacter sp.
Microbacterium sp.
Curvibacter sp.
Live Bd
M
Collimonas sp.
Collimonas sp. and J. lividum (str. BTP)
J. lividum (str. BTP)
Live Bd
N
Microbacterium sp.
Microbacterium sp. and Collimonas sp.
Collimonas sp.
Live Bd
O
Microbacterium sp.
Microbacterium sp. and J. lividum (str. BTP)
J. lividum (str. BTP)
Live Bd
